# Oxidative Stress: A Culprit in the Progression of Diabetic Kidney Disease

**DOI:** 10.3390/antiox13040455

**Published:** 2024-04-12

**Authors:** Na Wang, Chun Zhang

**Affiliations:** Department of Nephrology, Union Hospital, Tongji Medical College, Huazhong University of Science and Technology, Wuhan 430022, China

**Keywords:** diabetic kidney disease, oxidative stress, reactive oxygen species, molecular pathways, antioxidant therapy

## Abstract

Diabetic kidney disease (DKD) is the principal culprit behind chronic kidney disease (CKD), ultimately developing end-stage renal disease (ESRD) and necessitating costly dialysis or kidney transplantation. The limited therapeutic efficiency among individuals with DKD is a result of our finite understanding of its pathogenesis. DKD is the result of complex interactions between various factors. Oxidative stress is a fundamental factor that can establish a link between hyperglycemia and the vascular complications frequently encountered in diabetes, particularly DKD. It is crucial to recognize the essential and integral role of oxidative stress in the development of diabetic vascular complications, particularly DKD. Hyperglycemia is the primary culprit that can trigger an upsurge in the production of reactive oxygen species (ROS), ultimately sparking oxidative stress. The main endogenous sources of ROS include mitochondrial ROS production, NADPH oxidases (Nox), uncoupled endothelial nitric oxide synthase (eNOS), xanthine oxidase (XO), cytochrome P450 (CYP450), and lipoxygenase. Under persistent high glucose levels, immune cells, the complement system, advanced glycation end products (AGEs), protein kinase C (PKC), polyol pathway, and the hexosamine pathway are activated. Consequently, the oxidant–antioxidant balance within the body is disrupted, which triggers a series of reactions in various downstream pathways, including phosphoinositide 3-kinase/protein kinase B (PI3K/Akt), transforming growth factor beta/p38-mitogen-activated protein kinase (TGF-β/p38-MAPK), nuclear factor kappa B (NF-κB), adenosine monophosphate-activated protein kinase (AMPK), and the Janus kinase/signal transducer and activator of transcription (JAK/STAT) signaling. The disease might persist even if strict glucose control is achieved, which can be attributed to epigenetic modifications. The treatment of DKD remains an unresolved issue. Therefore, reducing ROS is an intriguing therapeutic target. The clinical trials have shown that bardoxolone methyl, a nuclear factor erythroid 2-related factor 2 (Nrf2) activator, blood glucose-lowering drugs, such as sodium-glucose cotransporter 2 inhibitors, and glucagon-like peptide-1 receptor agonists can effectively slow down the progression of DKD by reducing oxidative stress. Other antioxidants, including vitamins, lipoic acid, Nox inhibitors, epigenetic regulators, and complement inhibitors, present a promising therapeutic option for the treatment of DKD. In this review, we conduct a thorough assessment of both preclinical studies and current findings from clinical studies that focus on targeted interventions aimed at manipulating these pathways. We aim to provide a comprehensive overview of the current state of research in this area and identify key areas for future exploration.

## 1. Introduction

Diabetes mellitus (DM) is a complicated multisystemic metabolic disease featured by hyperglycemia, resulting from an insufficiency of insulin secretion or insulin resistance, which renders the body unable to respond fully to insulin. According to the 2021 International Diabetes Federation (IDF) Diabetes Atlas (10th edition), it was estimated that roughly 0.573 billion adults (aged 20–79 years old) globally were affected by DM, and the number was projected to escalate to 0.643 billion by 2030 and 0.783 billion by 2045 [1]. Sustained glucose dysregulation can result in macrovascular complications (including angina, coronary artery disease (CADs), myocardial infarction (MI), congestive heart failure (CHF), peripheral artery disease (PAD), and stroke) and microvasculopathy (including retinopathy, neuropathy, and nephropathy) [2]. Diabetic kidney disease (DKD), also called diabetic nephropathy (DN), is one of the main complications of DM. The prevalence of DKD has snowballed consistent with the burgeoning growth in the occurrence of DM. Approximately 30–40% of individuals with diabetes are diagnosed with DKD, which is the leading cause of chronic kidney disease (CKD), eventually developing end-stage renal disease (ESRD) and necessitating costly dialysis or kidney transplantation [3]. DM and its complications have become a severe public health and economic burden worldwide, especially in less developed countries [4,5,6]. Thus, there is still a vastly unmet medical need, making it imperative that we continue our search for innovative treatment targets.

DKD is caused by intricate interactions between multiple pathways, which are triggered by hyperglycemia and hemodynamic alterations associated with diabetes [7]. These alterations ultimately lead to albuminuria, a loss of kidney function, renal fibrosis, and inflammation. Oxidative stress is also widely recognized as a significant and integral factor that connects hyperglycemia with the vascular complications observed in diabetes, particularly DKD [8].

It is essential to mention that albuminuria and a decline in kidney function do not always develop concurrently. Traditionally, microalbuminuria or macroalbuminuria initiates the progression of DKD, with or without a progressive decline in the renal function as measured by the estimated glomerular filtration rate (eGFR) [9,10]. The main pathological alterations, including the thickening of tubular and glomerular basement membranes, the detachment of podocyte foot processes from the glomerulus, changes in the glycocalyx in endothelial cells, diffuse mesangial enlargement and sclerosis, increased apoptosis, and kidney interstitial fibrosis cumulatively contribute to the onset and progression of DKD [11]. Although extensive studies have been performed to comprehend the underlying mechanisms of DKD, the onset of DKD is occult and the complete pathogenesis of DKD remains elusive. Our finite knowledge of the pathogenesis of DKD results in limited therapeutic efficiency among the populations with DKD. The maintenance of optimal blood glucose and blood pressure levels is integral to preventing and managing DKD. Medications such as angiotensin-converting enzyme inhibitors (ACEIs) and angiotensin receptor blockers (ARBs), as well as sodium glucose cotransporter-2 inhibitors (SGLT-2i) and nonsteroid aldosterone receptor antagonists (NS-MRAs) have been approved for the purpose of slowing the progression of DKD [12]. Glucagon peptide-1 (GLP-1) agonists, which have cardioprotective effects, are promising agents to halt the development of DKD [12]. However, in spite of the emergence of these drugs, current therapies can only partially prevent the progression of DKD. It is imperative to delve deeper into the underlying mechanisms of DKD to develop more effective therapies that can curtail the overall disease burden. Although the clinical and experimental data indicate the role of the RAAS in driving DKD, the precise mechanisms behind these observations still require thorough exploration. Nevertheless, it has been established that angiotensin II possesses pro-oxidant properties, which can be attributed, at least partially, to its stimulation of nicotinamide adenine dinucleotide phosphate (NADPH) oxidase (Nox) [13]. GLP-1 receptor agonists (GLP-1 RAs) treatment has been shown to effectively reduce albuminuria, inflammation, and oxidative stress [14,15,16]. Additionally, SGLT2 inhibitors have been demonstrated to possess anti-inflammatory and oxidative stress-reducing properties [17], which extend beyond their blood glucose-lowering effects. 

Notably, hyperglycemia-induced glucose metabolism disorders primarily lead to the overproduction of superoxide and heightened oxidative stress in the kidney. It is noteworthy that this imbalance results in the damage and dysfunction of kidney cells, ultimately leading to the development of DKD. Multiple cytokines, the accumulation of advanced glycation end products (AGEs), and the activated polyol pathway can trigger oxidative stress [18], leading to lesions in the small vessels of the kidney and hastening kidney damage. Hyperglycemia, a primary culprit in the development of DKD, also serves as the primary instigator of renal tubular injury. Ample studies have established that hyperglycemia triggers oxidative stress by boosting the production of reactive oxygen species (ROS), which in turn promote the development of epithelial-to-mesenchymal transition (EMT) in renal tubular cells under high glucose conditions. An elevation in glucose levels enhances the formation of AGEs in bovine endothelial cells. This process can be mitigated through antioxidant treatment [19]. Furthermore, the interaction between AGEs and the receptors for AGEs (RAGE) in human endothelial cells resulted in the production of ROS, including hydrogen peroxide, through mechanisms involved in the activation of NADPH oxidases-2 (Nox2) [20]. In diabetic kidneys, the overproduction of ROS is primarily due to the hyperactivity of Nox enzymes and mitochondrial dysfunction [13]. This dysfunction is often accompanied by an inadequate antioxidant system, leading to a further surge in ROS production. The excessive generation of ROS and the subsequent collapse of antioxidant defense mechanisms play pivotal and critical roles in the development and progression of DKD [8]. 

In this review, we comprehensively review the accumulating evidence that points to the involvement of heightened oxidative stress and compromised antioxidant defense mechanisms in the pathophysiology of DKD. Furthermore, we delve into recently developed drugs that aim to treat DKD by targeting oxidative stress. Research and review papers were identified through a “pearl-growing” approach, citation chasing, and a search of PubMed, using terms related to “diabetic kidney disease” or “diabetic nephropathy” and “oxidative stress” and “pathophysiology” and “therapeutic targets”. The primary focus was on research and review papers published within the past decade.

## 2. Sources of Endogenous ROS

Oxidative stress is the result of a disruption in the balance between oxidants and antioxidants. Oxidative stress occurs when the production of ROS overwhelms the quenching capacity of antioxidants. Hyperglycemia boosts the production of ROS, which can initiate a chronic inflammatory response. Furthermore, this oxidative stress and persistent inflammation can feed into each other, enhancing hyperglycemia and insulin resistance through various mechanisms, including the disruption of insulin signal transduction (IST) and the enhancement of β-cell and mitochondrial dysfunction. The insulin resistance in HepG2 cells can be improved by elevating the expression levels of nuclear factor erythroid 2-related factor 2 (Nrf2), superoxide dismutase (SOD), NADPH quinone oxidoreductase (NQO1), heme oxygenase-1 (HO-1), and glutathione peroxidase (GSH-Px) [21]. This can be achieved by downregulating the levels of malondialdehyde (MDA), ROS, and c-Jun N-terminal kinases (JNKs). Persistent inflammation can ultimately result in renal endothelial dysfunction and various vascular complications. The accumulation of ROS, including superoxide, singlet oxygen, alpha oxygen, hypochlorous acid, and hydroxyl radicals, can result in the direct oxidation of vital biomolecules, including carbohydrates, lipids, proteins, and DNA [22]. This oxidation can disrupt signal transduction pathways and cause cell dysfunction, ultimately leading to cell death. This imbalance can lead to serious damage to cells and tissues, ultimately leading to various chronic diseases and aging. The reduction in hyperglycemia-induced overproduction of ROS resulted in enhanced endothelium-dependent vasodilation in the aortas of mice that were fed a high-fat diet (HFD) and administered streptozotocin (STZ) to induce diabetes [23]. This finding highlights the importance of maintaining a balance between oxidants and antioxidants to preserve cellular health and prevent the development of chronic diseases and aging.

**Figure 1:**
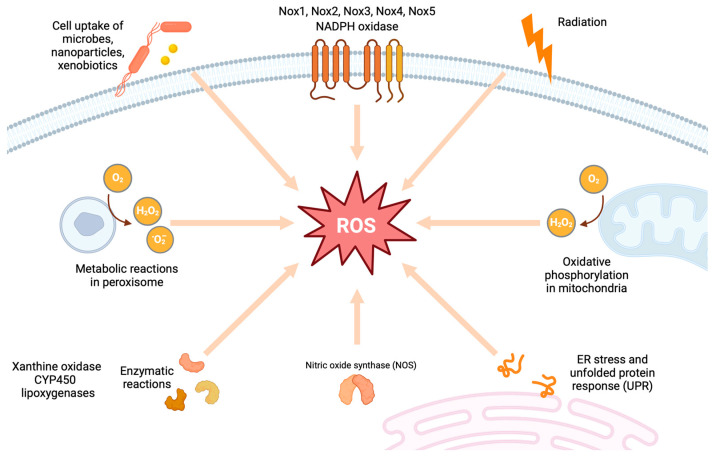
Endogenous sources of reactive oxidative species (ROS). Endogenous sources of ROS include mitochondrial ROS production, NADPH oxidases (Nox1, Nox2, Nox3, Nox4, and Nox5), uncoupled endothelial nitric oxide synthase (eNOS), xanthine oxidase (XO), cytochrome P450 (CYP450), and lipoxygenase.

Common sources of endogenous ROS include mitochondrial ROS production, Nox, uncoupled endothelial nitric oxide synthase (eNOS), xanthine oxidase (XO), cytochrome P450 (CYP 450), and lipoxygenase (Figure 1). Upon stimulation with adverse factors from both the internal and external environments, numerous reactive oxygen and nitrogen radicals are generated within the body, which cannot be completely neutralized by the antioxidant defense system. This leads to a range of physiological and pathological reactions in cells and tissues. Additionally, the intricate interplay between various factors, including irregularities in glycolipid metabolism and hemodynamic alterations, triggers processes like polyol and hexosamine pathways, which significantly boost ROS production. ROS can function as second messengers in various signaling cascades and also serve as regulators that modulate metabolism, as well as the activation, proliferation, differentiation, and apoptosis of immune cells [24]. This imbalance in the oxidant–antioxidant equilibrium in the kidneys subsequently triggers oxidative stress responses. As a consequence, downstream cellular signaling cascades are activated, leading to a series of adverse events, such as inflammation, autophagy, and fibrosis. These processes accelerate the progression of pathological changes and functional abnormalities, ultimately leading to the development of DKD [13].

### 2.1. Mitochondrial Superoxide Production

The mitochondria are the energy-producing powerhouses of eukaryotic cells, playing a vital role in providing energy to cells that demand high levels of energy. The mitochondria, particularly, but also to a lesser extent, the cytoplasm, are the primary intracellular sources of oxidants. Superoxide (O_2_^•–^) and hydrogen peroxide (H_2_O_2_) are the most prevalent oxidant species (Figure 1). Within the mitochondria, oxidative phosphorylation (OXPHOS), the uncoupling of the respiratory chain, and the dysregulation of complex I, coenzyme Q, and complex III are the primary sources of ROS production [25]. The electron transport chain (ETC) and OXPHOS are the mitochondria’s primary mechanisms for generating energy. Some studies have shown that mitochondrial superoxide production in the liver, heart, and skeletal muscle tissue remains unchanged in the presence of diabetes [26]. However, other studies consistently indicate that, in numerous sites affected by diabetes-related complications, mitochondrial ROS levels in rat kidneys are elevated, and changes in mitochondrial bioenergetics and dynamics may accelerate the progression of DKD, albeit not always in a consistent manner [27]. This indicates that mitochondrial dysfunction is postulated to be a potential underlying cause of DKD.

When hyperglycemia occurs, the citric acid cycle generates increased amounts of reduced nicotinamide adenine dinucleotide (NADH) and reduced flavin adenine dinucleotide (FADH2). In addition, the ETC generates a substantial reducing force, which can result in electron leakage. This leakage combines with oxygen molecules to produce ROS, ultimately resulting in oxidative stress. Rotenone, a specific inhibitor of mitochondrial complex-I, has been found to increase the proportions of glutathione and NADPH [28]. This modulation of the redox system not only rectifies the imbalance but also reduces fibrosis, inflammation, and oxidative damage. Furthermore, it attenuates glomerular and tubular injuries in mice with STZ-induced diabetes. Mitochondrial fission and fusion, also known as mitochondrial dynamics, can influence cell function. In particular, when hyperglycemia is present, an increase in mitochondrial fission and a decrease in mitochondrial fusion with renal cells suggest the mitochondrial dynamics have been altered [29]. The alterations in mitochondrial bioenergetics and dynamics precede the onset of albuminuria and renal histological damage in diabetic rats [27]. In addition to the heart, the kidneys also exhibit exceptionally high rates of oxygen consumption and mitochondrial activity. In kidneys with high energy demands, the presence of numerous dysfunctional mitochondria can contribute to the development of DKD [30]. Consequently, alleviating mitochondrial dysfunction may offer a promising non-traditional treatment approach.

### 2.2. NADPH Oxidases (Noxs)

Noxs are widely recognized as “professional ROS producers” as their primary function of generating ROS (Figure 1). After mitochondria, Noxs are the primary sources of ROS in the kidneys. In contrast to other enzymes that produce ROS as a by-product of their enzymatic activity, these enzymes are unique in their ability to directly generate ROS [13]. In normal physiological environments, these enzymes remain relatively inactive. However, when exposed to a high glycemic environment, they become activated, leading to an increase in their transcription and translation. There are several isoforms of Nox enzymes, including Nox1, Nox2, Nox3, Nox4, and Nox5, as well as dual oxidases 1 and 2 (DUOX1 and DUOX2). These isoforms exhibit distinct patterns of organ-specific expression and vary in their composition, mode of activation, and the type of ROS they produce. While most Nox isoforms predominantly produce superoxide, Nox4 primarily generates H_2_O_2_. In previous studies, Nox1 has been identified as a key player in vascular injury in diabetes, particularly in retinopathy. However, its involvement in DKD remains unexplored [13]. Nox2, on the other hand, is significantly upregulated in individuals with diabetes and is also highly expressed in immune cells, including phagocytes and macrophages [13]. Certainly, STZ-induced diabetic Nox2 knockout mice exhibited a significant increase in mortality, primarily due to severe infections [31]. Nox3 expression is known to rise with cisplatin exposure, aging, and noise insults, specifically in certain cell types within the cochlea [32,33]. This upregulation leads to the apoptotic loss of outer hair cells, making Nox3 a potential molecular target for the development of therapeutics aimed at treating sensorineural hearing loss, particularly cisplatin-induced, age-related, and noise-induced hearing loss. Nox3 has been associated with diabetic nephropathy and/or renal damage in models of hypertension [34]. It is thought that Nox3 plays a role in the development of renal damage in individuals with diabetes and hypertension; however, further research is needed to understand its precise mechanisms and potential therapeutic targets. In DKD, Nox4 is the dominant isoform found in the kidneys. Nox4 was initially named “Renox” due to its high constitutive activity in the kidney, its upregulation in diabetes, and its association with increased ROS production and renal injury [35]. The genetic manipulation or pharmacological inhibition of Nox4 effectively alleviates renal protection in the setting of long-term diabetic nephropathy induced by STZ administration in ApoE(−/−) mice [35]. When there is an increase in ROS production and a high glucose level, the overexpression of Nox4 can lead to mesangial expansion and glomerular hypertrophy.

The significance of Nox4 expression and its associated ROS production in podocyte injury during diabetes was clearly established through using a podocyte-specific Nox4 knockout mouse model rendered diabetic via STZ injections [36]. This study revealed several noteworthy observations. Firstly, the mice exhibited a significant reduction in albuminuria, a key indicator of kidney function. Secondly, glomerular basement membrane thickness, an essential structural component of the kidney, was found to be reduced. Finally, electron microscopy analysis revealed a reduction in foot process effacement, a pathological feature associated with podocyte injury. These findings underscore the core role of Nox4 expression and ROS production in podocyte injury during diabetes. In contrast to the kidney, where Nox4 deletion is renoprotective, the role of Nox4 in the macrovasculature remains controversial. While some studies suggest a vasculoprotective effect of Nox4, others have found that the global deletion of Nox4 is associated with increased atherosclerosis development in a diabetic atherosclerosis model [37]. Therefore, further research is required to clarify the precise role of Nox4 in the macrovasculature and its potential impact on cardiovascular disease. Moreover, transforming growth factor beta 1 (TGF-β1) enhances the expression of early growth response factor-1 (EGR1) in keloids by modulating the small mother against decapentaplegic (SMAD) pathway, thereby promoting the fibrotic phenotype of keloid fibroblasts [38]. EGR1, in turn, regulates the production of ROS by targeting Nox4. Consequently, ROS derived from Nox4 enhances the fibrotic phenotype of keloid fibroblasts, playing a crucial role in keloid fibrosis. The TGF-β1/EGR1/Nox4 axis may act as a potential therapeutic target for the management of fibrosis related disease. The role of Nox5 in humans remains enigmatic. In transgenic mice where Nox5 was specifically expressed in podocytes, there was a notable upsurge in albuminuria and renal injury, even in the absence of diabetes [39]. This increase was exacerbated when diabetes was introduced into the mice. Conversely, in diabetic models with a selective expression of Nox5 in vascular smooth muscle cells, including mesangial cells, there was a significant augmentation in renal fibrosis and mesangial expansion, with relatively minor effects on albuminuria and inflammation [40,41]. Moreover, accumulating evidence points to an elevated expression of Nox5 in the human kidney in DKD [39], as well as in the vasculature [42], the eye [43], and nerves [44]. This suggests that the increased activation of Nox5 is detrimental at multiple organ sites. Therefore, more exhaustive research is necessary to comprehend the exact function of Nox5 in the context of multiple diabetic complications.

### 2.3. Uncoupled NOS

The eNOS utilizes l-arginine to generate NO, a process that appears to be heightened in the early stages of DKD (Figure 1). However, as the disease progresses, the production of NO seems to decrease [45]. Glu298Asp polymorphism in the eNOS gene has been shown to modulate NO production and has been associated with DKD [46]. Decreased soluble guanylate cyclase activity and cyclic guanosine monophosphate (cGMP) levels in diabetic kidneys were shown to influence the progression of nephropathy through modulating the protective actions of NO [47,48]. Therefore, drugs capable of restoring guanylate cyclase activity and cGMP levels could potentially offer a promising adjunctive therapeutic approach for individuals with diabetic nephropathy. In agreement with the above findings, when eNOS is deficient in the db/db mouse model of type 2 diabetes, it results in a significant exacerbation of diabetic kidney damage, as evidenced by mesangial expansion, glomerular basement membrane thickness, and increased albuminuria [49]. Additionally, the absence of l-arginine or the essential cofactor tetrahydrobiopterin (BH4) causes eNOS to become uncoupled, resulting in the production of superoxide instead of NO [50]. The uncoupling of eNOS plays a notable role in the overproduction of ROS, both in vitro under conditions of high glucose stimulation, and in vivo in mouse models of diabetes, including those models specifically designed to study DKD [50]. Interestingly, angiotensin (AT1) receptor blockade and the inhibition of 3-hydroxy-3-methylglutaryl-coenzyme A (HMG-CoA) reductase have been observed to normalize the diabetes-induced reduction in the expression of the BH4 synthesizing enzyme GTP cyclohydrolase I [51,52]. These interventions not only promote the recoupling of eNOS, but also inhibit Nox, thereby contributing to the restoration of endothelial function.

### 2.4. Xanthine Oxidase (XO)

The enzyme XO is primarily responsible for the synthesis of uric acid and superoxide anions in the liver. The enzyme XO catalyzes the conversion of hypoxanthine, a by-product of purine degradation, into xanthine. Subsequently, xanthine is oxidized to uric acid, generating ROS as a by-product of these reactions (Figure 1). It is worth noting that higher concentrations of uric acid are associated with an increased risk of developing DKD [53]. The highly activated XO elevated the level of intracellular ROS, which resulted in renal damage due to direct oxidative damage to renal cells [54]. Additionally, it indirectly triggered inflammatory responses by activating the nuclear transcription factor B (NF-κB) signaling pathway. Previous research revealed that acetate effectively mitigated diabetes-induced nephrotoxicity by suppressing XO and its proinflammatory mediators [55]. Allopurinol, an inhibitor of XO, effectively lowers uric acid levels, diminishes albuminuria, and mitigates tubulointerstitial injury. However, it fails to prevent mesangial expansion, which is attributed to a reduction in intercellular adhesion molecule 1 (ICAM-1) expression by tubular epithelial cells in type 2 diabetic db/db mice [56]. Surprisingly, allopurinol does not alleviate oxidative stress in the kidney. The effect of the XO inhibitor febuxostat on type 2 diabetic Zucker obese (ZO) rats was also evaluated over an 18-week period. Febuxostat successfully normalized serum uric acid levels and mitigated the progression of albuminuria, renal structural changes, and the expression of TGF-β, connective tissue growth factor (CTGF), collagen 4, mesenchymal markers (ferroptosis suppressor protein-1 (FSP1) and vimentin), and nitrotyrosine, a tissue marker of oxidative stress in the kidneys [57]. Moreover, recognizing the role of ROS as secondary messengers in healthy cells is essential, as their presence at low and relatively constant levels is vital for maintaining normal cellular homeostasis [58]. The primary objective of antioxidant treatment strategies is to curtail the overproduction of oxidants and also to counteract those that have already been generated. Nevertheless, factors such as precise timing, localization, and the extent of oxygen scavenging are of paramount importance when implementing this approach as a therapeutic intervention.

### 2.5. Cytochrome P450

The cytochrome P450 system plays a pivotal role in enhancing hyperglycemia-induced ROS generation through the Nox family of enzymes (Figure 1). Notably, CYP4A protein expression was elevated in the isolated microsomes of podocytes cultured under high glucose conditions. The upregulation of CYP4A in response to high glucose stimulation was accompanied by a significant increase in the production of its metabolite, 20-hydroxyeicosatetraenoic acid (20-HETE). This metabolite was found to be associated with elevated ROS production, which was linked to the activation of Nox enzymes. CYP450 epoxygenases are capable of converting arachidonic acid into epoxyeicosatrienoic acids (EETs), which play significant and diverse roles in the cardiovascular system [59]. EETs possess anti-inflammatory, anti-apoptotic, pro-angiogenic, and anti-hypertensive properties within the cardiovascular system, indicating a potential therapeutic role in the development of DKD. The research indicates that glomerular injury caused by hyperglycemia is triggered by a decrease in cytochrome P450 2C11 (CYP2C11)-derived EET formation [60]. This reduction in EETs subsequently leads to the activation of vascular endothelial growth factor A (VEGF-A) signaling and an increase in Nox4 expression. In the kidney, 20-HETE, a primary eicosanoid derived from cytochrome P450 4 (CYP4), potentiates the constriction of renal vessels, ultimately leading to hypertension [61]. Additionally, it triggers renal tubular cell hypertrophy and podocyte apoptosis, further contributing to the development of hypertension and renal dysfunction. Hypertension and podocyte injury play a fundamental role in the development of DKD and serve as significant predictors of disease progression. In the OVE26 mouse model of type 1 diabetes, studies further revealed that the generation of ROS by 20-HETE, CYP4A monooxygenases, and Nox oxidases was associated with albuminuria, podocyte loss, and podocyte effacement [62]. The CYP4A inhibitor, N-hydroxy-N′-(4-butyl-2-methylphenol) formamidine (HET0016), effectively prevented podocyte apoptosis and mitigated proteinuria in this model. These findings suggest that these pathways play a significant role in the pathogenesis of DKD and may provide potential targets for therapeutic intervention.

### 2.6. Lipoxygenase

Lipoxygenases (LOXs) are enzymes that metabolize arachidonic acid into HETEs. According to numerous studies, increased 12/15-LOX activity and 12-HETE production contribute to heightened renal oxidative stress, inflammation, and injury in STZ-induced diabetic mice [63] (Figure 1). It has also been proposed that HETE may collaborate with angiotensin II and TGF-β to trigger fibrotic modifications in diabetic kidneys [64]. Furthermore, studies indicated that higher levels of LOX metabolites and lower levels of CYP450 metabolites were significantly associated with the development of DKD in patients with T2D [65]. This suggests that LOX and CYP450 metabolites could potentially serve as therapeutic targets for preventing or treating DKD with T2D.

## 3. The Pathogenesis of Diabetic Kidney Disease (DKD) with Oxidative Stress

**Figure 2:**
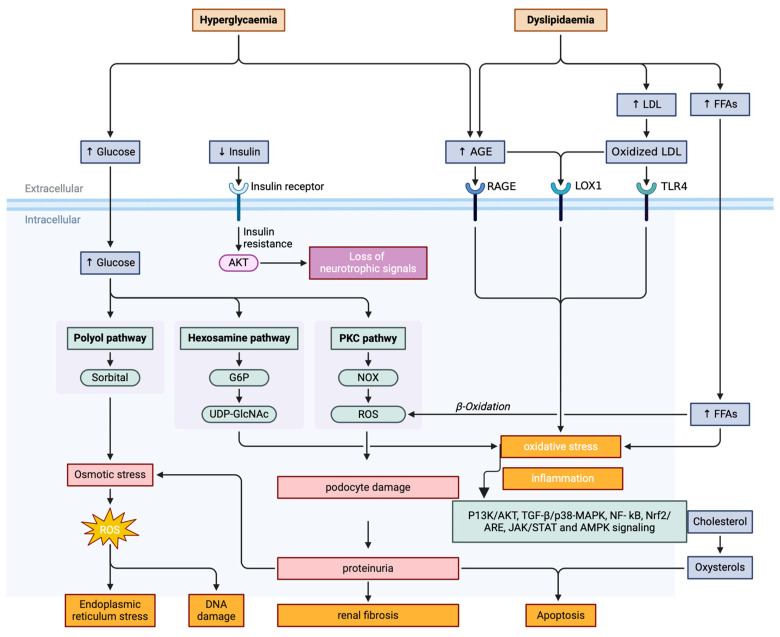
Pathogenesis of diabetic kidney disease (DKD) with oxidative stress. G6P: glucose-6-phosphate; UDP-GlcNAc: uridine diphosphate N-acetylglucosamine; AGEs: receptor for advanced glycation end products; RAGEs: receptor for advanced glycation end products; LOX1: lipoxygenase 1; LDL: low-density lipoprotein; TLR4: toll-like receptor 4; FFAs: free fatty acids; PI3K/Akt: phosphoinositide 3-kinase/protein kinase B; TGF-β/p38-MAPK: transforming growth factor beta/p38-mitogen-activated protein kinase; NF-κB: nuclear factor kappa B; Nrf2/AREs: nuclear factor erythroid 2-related factor 2/antioxidant response elements; JAK/STAT: Janus kinase/signal transducer and activator of transcription; AMPK: adenosine monophosphate-activated protein kinase. These reactions promote inflammation, fibrosis, and apoptosis, ultimately intensifying the progression of DKD.

During the development of DKD, oxidative stress assumes a pivotal role. Stress is caused by an excessive production and/or a decrease in the capacity to eliminate ROS in the body. The accumulation of ROS can directly cause oxidative damage to cellular components, ultimately leading to a series of pathophysiological changes (Figure 2). Not only does oxidative stress cause direct damage to cells, but it also triggers inflammatory responses through the activation of the NF-κB signaling pathway. The activation of NF-κB can further enhance the production of ROS, creating a vicious cycle that intensifies kidney damage.

Hyperglycemia and its by-products, including AGEs, as well as secondary mediators like angiotensin II and aldosterone, contribute to the injury and dysfunction of renal parenchymal cells. These resident renal cells trigger cellular and molecular mechanisms that lead to persistent renal inflammation, ultimately resulting in long-term chronic inflammation [66]. The innate immune system plays a particularly critical role in the response to inflammation. Inflammatory cells may also produce excessive ROS under pathological or proinflammatory conditions. A previous single-cell transcriptomic analysis has revealed a significant increase in the infiltration of leukocytes into the kidneys of individuals with early DKD, ranging from 7- to 8-fold higher compared to healthy controls [67]. These findings suggest a robust immune response in the kidneys of individuals with early DKD, which may play a role in the development and progression of the disease.

### 3.1. Immune Cells

Macrophages, the primary immune cells, show a key effect on the development and progression of DKD. Macrophages were found to infiltrate the glomeruli and interstitium in renal biopsy samples from patients with all stages of DKD [68]. This infiltration is strongly linked to the development of interstitial fibrosis and the subsequent increase in serum creatinine levels [69]. During the initial stages of DKD, proinflammatory M1 macrophages invade the renal parenchyma. Once activated, M1 macrophages can produce ROS and proinflammatory cytokines, including interleukin-1 (IL-1), interleukin-6 (IL-6), matrix metalloproteinase 9 (MMP 9), and tumor necrosis factor α (TNF-α) [70]. However, as the disease progresses, these macrophages can transition to the profibrotic M2 phenotype. The involvement of macrophages in DKD is complex and involves various signaling pathways that can interact and cross-talk, further complicating the development of effective treatment strategies. Therefore, a more exhaustive understanding of the role of macrophages in DKD is necessary to identify potential therapeutic targets and approaches that can effectively manage this condition. Given the intricate nature of macrophage-targeted strategies for the treatment of DKD, it is essential to conduct more in-depth preclinical studies and clinical trials to better understand their potential and limitations. This will aid in the development of effective treatment strategies that can effectively manage DKD and improve the quality of life of patients.

Neutrophils are the most prevalent circulating immune cells in the body, playing a key role in immune response. Studies on patients with autoimmune diabetes have shown that those with macroalbuminuria, as opposed to normoalbuminuria, exhibit significantly higher serum neutrophil counts. Additionally, a parallel study involving patients with T2DM reported a significant correlation between the neutrophil-to-lymphocyte ratio (NLR) and both DKD and albuminuria [71]. High levels of these ratios may serve as predictive and prognostic markers for the development of DKD. The ROS generated by Noxs seem to play an important role in the formation of neutrophil extracellular traps (NETs) [72]. The activation of NADPH oxidase is integral to the NET process, which is triggered by elevated glucose levels. Neutrophils are the first responders recruited to sites of inflammation and contribute to host defense activity through phagocytosis, degranulation, and the extrusion of NETs [73]. A significant increase in NET deposition in glomeruli was observed in both DKD patients and diabetic mice, whether induced by streptozotocin or the db/db genotype. After treating diabetic mice with DNase I to degrade NETs, studies revealed a noticeable improvement in glomerulopathy and a reduction in glomerular endothelial cell (GEC) injury [74]. Neutrophils play a central role in the pathogenesis of DKD. NETs, which are webs of DNA and proteins released by neutrophils, have been shown to promote the NOD-like receptor family, pyrin domain containing 3 (NLRP3) inflammasome activation, an imperative step in the development of DKD. This activation results in the release of proinflammatory cytokines that contribute to glomerular endothelial dysfunction, a hallmark of DKD [75]. Targeting NETs may be a potential therapeutic strategy to mitigate the progression of this debilitating disease.

Mast cells are frequently observed in fibrotic lesions of various organs and tissues, indicating their potential involvement in the development of fibrosis. In normal kidneys, mast cells are either scarce or absent, but in kidneys affected by fibrosis due to primary diseases such as DKD, IgA nephropathy, membranous nephropathy (MN), allograft fibrosis, and chronic cyclosporin toxicity, mast cells are abundant [76]. The degranulation of mast cells releases a range of mediators, including inflammatory cytokines, endothelins, growth factors, and proteolytic enzymes [77]. Furthermore, there is a notable increase in the renal density of mast cells in diabetic patients suffering from DKD. The study revealed that mast cells play a pivotal role in the development of DKD by releasing bioactive substances, including tryptase, chymase, TGF-β1, renin, and TNF-α, through degranulation [78]. These mast cells promote renal inflammation and fibrosis, ultimately leading to the progression of diabetic nephropathy. Chymase, a serine protease, is located in the granules of mast cells. Chronic chymase inhibition decelerated the progression of urinary albumin excretion in the diabetic mice [79]. Previous research has established the negative impact of mast cells in DKD, while others argue that mast cells can indeed be protective [80]. To develop effective therapeutic agents with different targets, such as the infiltration and activation of mast cells, more preclinical studies are essential prior to clinical application.

Type 2 diabetic patients exhibited a notable surge in CD4^+^, CD8^+^, and CD20^+^ cells within the interstitium [81]. Notably, the quantities of CD4^+^ and CD20^+^ cells were directly linked to the severity of proteinuria. The study provided clear evidence that the abnormal infiltration of immune cells into the kidney and the activation of T cells within the interstitium were the underlying immunopathological mechanisms of diabetic kidney injury. The reprogramming of metabolism is a key process in the activation of T cells [82]. Specifically, the switch from oxidative phosphorylation to glycolysis as the primary energy source results in an elevation of NADPH levels. ROS play a prominent role in T-cell hyporesponsiveness, apoptosis, and activation [83]. Additionally, mitochondrial dysfunction is the primary source of oxidative stress in T cells. Tregs have the potential to mitigate the pathogenic processes of DKD [84]. The imbalance between Th17 and Treg cells displays a profound influence on the development of DKD. Dapagliflozin can lead to the attenuation of albuminuria and tubulointerstitial fibrosis by inhibiting serum/glucocorticoid-regulated kinase 1 (SGK1) and correcting the imbalance between Th17 and Treg cells [85]. The accumulating data from experimental models of DKD reveal a rise in the number of T cells in the bloodstream and kidney cortex. This increase subsequently triggers the secretion of inflammatory mediators, like interferon-γ (IFN-γ) and TNF-α, which further activate cells in the innate immune response [86]. Human studies have also shown that the number of T cells present in the kidney’s interstitial region is directly linked to the severity of albuminuria in individuals with type 2 diabetes. Furthermore, it is essential to acknowledge that there might be disparities in the T cells present in the blood and kidneys of individuals with DKD. This underscores the importance of examining the T-cell-receptor milieu in the kidneys. Future research efforts aiming to develop innovative therapeutic approaches for DKD should focus on the fundamental mechanisms of T-cell infiltration and activation.

B cells are also implicated in the pathogenesis of T1D, playing a role in the autoimmune destruction of pancreatic β cells, which is primarily mediated by T cells. The selective depletion of B lymphocytes using rituximab, an anti-CD20 monoclonal antibody, significantly decelerated the decline in β-cell function in patients with recent-onset T1D after one year of treatment [87]. Rituximab delays the decline in C peptide, yet it does not appear to significantly alter the underlying pathophysiology of the disease. The count of circulating CD19^+^CD24^hi^CD38^hi^ B cells is directly proportional to the levels of eGFR and serum interleukin-10, but inversely related to urinary protein levels in patients with diabetic nephropathy [88]. Regulatory B cells seem to play a protective role in DKD and might serve as a potential therapeutic target in the treatment of DKD. 

### 3.2. Complement System

The complement system, the essential and fundamental component of innate immune responses, actively participates in the development of autoimmune kidney diseases, diabetic kidney disease, and focal segmental glomerulosclerosis. The complement system is essential for immune cells activation and infiltration, leading to renal parenchymal cell damage, and the subsequent deterioration of renal function [89]. Once activated, the complement system promptly produces substantial amounts of protein fragments, which act as powerful mediators in inflammatory, vasoactive, and metabolic responses [90]. Complement plays a pivotal role in host defense activity and maintaining homeostasis; however, its inappropriate or uncontrolled activation can lead to tissue damage. It is evident that mannose-binding lectin (MBL), H-ficolin, the complement component C3, and the membrane attack complex (MAC) all potentially contribute to renal injury in a hyperglycemic environment [91]. 

In addition, the research also revealed that the levels of urinary interferon-γ (INF-γ) and MBL served as significant independent risk factors for the development of DKD in patients with T2DM [92]. Complement 7 was recognized to be linked to early diabetic nephropathy (EDN), making it a potential target for both detecting and treating EDN [93]. Furthermore, the deposition of C1q, C3c, and C4c on renal histopathology was strongly associated with the severity of kidney damage in patients with DKD [94,95,96]. The glomerular deposition of complement C3a was vital in podocyte injury, ultimately resulting in proteinuria and renal dysfunction in mice with type 2 diabetes [97]. Furthermore, clinical data revealed that the urinary levels of C3a and C5a were strongly associated with serum creatinine, urinary protein, and eGFR [98]. In contrast, the urinary levels of sC5b-9 were significantly connected to the latter two factors, but not to serum creatinine. Additionally, it was observed that the urinary levels of MBL, Bb, and C4d were significantly linked to urinary protein. Additionally, C3a, C4d, and Bb were significantly associated with the classification of glomerular lesions in DKD. 

In a study of 191 patients with type 1 diabetes, it was found that individuals with elevated levels of MBL were more prone to suffering from proteinuria [99]. Additionally, a study of 326 patients with T2D over a 15-year period revealed that higher levels of MBL were significantly associated with an increased risk of both death and the development of albuminuria [100]. The FinnDiane study convincingly demonstrated that the levels of MBL were linked to the progression from macroalbuminuria to ESRD in individuals with type 1 diabetes [101]. These observations suggest that MBL may play a role in the pathogenesis of DKD.

In patients with DKD, the complement system was activated, and among the three potential complement pathways, the activation of the lectin and alternative pathways was closely linked to renal damage [98,102]. Hyperglycemia is believed to activate the lectin pathway due to the glycation of pattern recognition molecules [102] and complement regulatory proteins, causing the uncontrolled activation of the complement system [103]. Blocking C3a and C5a receptors (C3aR/C5aR) could effectively alleviate EMT in DKD by inhibiting the Wnt/β-catenin signaling pathway [104]. The genetic ablation of C5aR1 in mice provided resistance against diabetes-induced renal damage [105]. The complement C5a/C5aR1 axis propagated renal injury in DKD by disrupting mitochondrial metabolic flexibility [105]. C3aR antagonism was shown to preserve podocyte number and prevent both proteinuria and kidney function decline in ob/ob mice with type 2 diabetes (monogenic obesity) [97]. In obese rats, the application of C3aR and C5aR antagonists was found to diminish the expression of proinflammatory adipokines and other inflammatory genes within adipose tissue [106]. 

While glycated C3 and C4 appeared early in diabetic patients, their function remained unaffected [107]. However, the glycation of CD59, a key regulator of the complement system, has been shown to result in a loss of its ability to inhibit the MAC, ultimately leading to endothelial cell damage [108]. The upregulation of CD59 may protect against MAC-mediated cell lysis and complement-mediated damage. In conclusion, the enhancement of CD59 expression can serve as a potential strategy to hinder the terminal pathway of complement activation. A clinical trial revealed that glycated CD59 was independently and positively associated with HbA1c levels, regardless of whether the individual had diabetes or not [109]. This finding suggests that CD59 glycation could be a valuable biomarker for monitoring diabetes control and a potential therapeutic target for treating diabetic complications. 

Diabetic nephropathy in non-obese diabetic (NOD) mice was characterized by the infiltration of T and B cells, along with CD11c^+^ dendritic cells, which had close contact with CD4^+^ and CD8^+^ T cells within the affected tissues [110]. In diabetic NOD mice, IgG deposits were observed in the glomeruli, accompanied by the presence of complement C3. This observation suggests a potential involvement of the immune system, specifically IgG and complement C3 in the development of diabetic nephropathy. Animal studies revealed that the absence of MBL could mitigate renal changes in an STZ-induced model of type 1 diabetes in mice [111]. This finding suggests that MBL deficiency may play a protective role in the development of diabetic nephropathy. 

### 3.3. The Upstream Signaling Cascades That Trigger Oxidative Stress Response

#### 3.3.1. AGEs/RAGE Pathway

AGEs are the highly reactive and irreversible end products of non-enzymatic reactions between glucuronyl and free amino groups, particularly those found in lipid and protein components. Persistent hyperglycemia and dyslipidemia stimulation can accelerate the production of AGEs. These excessive AGEs, in turn, have the potential to directly enhance the production of ROS. It is worth noting that the generated ROS will feedback to stimulate the production of AGEs, thus intensifying oxidative stress and ultimately causing renal tissue damage. Substantial research has indicated that hyperglycemia and dyslipidemia stimulation lead to an increased expression of RAGE in glomerular epithelial cells, mesangial cells, endothelial cells, and podocytes. The binding of RAGE to AGEs triggers NADPH oxidase activation, leading to a significant surge in ROS production in endothelial cells. This in turn disrupts molecular conformation and alters enzyme function, ultimately provoking oxidative stress responses. The interaction between AGEs and RAGE triggers multiple signaling cascades, including mitogen-activated protein kinase/extracellular signal-regulated kinase (MAPK/ERK), TGF-β, JNK, and NF-κB [112]. This activation results in heightened oxidative stress and inflammation, contributing to renal injury, such as mesangial expansion and renal interstitial fibrosis (Figure 2). Furthermore, the downstream effects of the AGEs/RAGE axis include disrupted insulin signaling, metabolic imbalance, RAGE-induced pancreatic beta-cell damage, and epigenetic modifications [112]. 

The inhibitor of AGE formation, aminoguanidine, was found to significantly reduce albuminuria, structural damage, macrophage infiltration, TGF-β1 expression, and collagen deposition in diabetic apolipoprotein E-knockout mice [113]. Notably, both pyridoxamine and aminoguanidine exhibited comparable efficacy in halting the progression of renal disease in diabetic rats [114]. Furthermore, these agents contributed to a reduction in hyperlipidemia and alleviated redox imbalances that were evident in these rats. In addition to aminoguanidine, another AGE inhibitor, alagebrium, was also found to effectively curtail glomerular fibrogenesis and inflammation beyond the prevention of RAGE activation in diabetic apolipoprotein E knockout mice [115]. Moreover, neutralizing murine RAGE with a specific antibody significantly reduced albuminuria and mitigated glomerular injury in obese type 2 diabetic mice [116]. These findings underscore the complexity of DKD and highlight the potential of AGE/RAGE blockades as therapeutic strategies for managing DKD.

#### 3.3.2. Hexosamine Pathway

The hexosamine pathway is a biochemical pathway that plays an eminent role in the regulation of glucose metabolism and insulin sensitivity. During glycolysis, approximately 2–5% of glucose-6-phosphate (G6P) is converted into fructose-6-phosphate (F6P) and subsequently enters the hexosamine pathway. This pathway is involved in the production of uridine diphosphate N-acetylglucosamine (UDP-GlcNAc), which is a vital substrate for the synthesis of proteoglycans and glycoproteins. The hyperactivation of the hexosamine pathway results in the synthesis of UDP-GlcNAc (Figure 2). Notably, GlcNAc has the ability to inhibit the phosphorylation of Akt (protein kinase B)/eNOS and heat shock protein (HSP) 72 through kinase-like serine/threonine phosphorylation [117]. This process further enhances the expression of TGF-β and plasminogen activator inhibitor-1 (PAI-1), ultimately leading to oxidative stress and fibrosis in DKD. The rate-limiting enzyme in this pathway is glutamine fructose-6-phosphate-amidotransferase (GFAT), which plays an essential function in regulating the amount of glucose. The hexosamine pathway is activated by elevated levels of glucose and insulin and is thought to contribute to the development of insulin resistance and type 2 diabetes. It is reported that hexosamine can trigger endoplasmic reticulum (ER) stress in endothelial cells and macrophages, as well as extracellular matrix (ECM) expression and apoptosis, ultimately resulting in heightened oxidative stress responses [118]. The inhibition of GFAT or other enzymes in the hexosamine pathway may provide a novel therapeutic approach for the treatment of DKD.

#### 3.3.3. Polyol Pathway

The activity of the polyol pathway in the kidney is exceptionally high, with the concentration of enzymes aldose reductase (AR), a key enzyme, peaking in the kidney medulla at 29.3 micrograms per milligram of protein. This elevated activity results in heightened oxidative and osmotic stress in the kidneys [18] (Figure 2), potentially causing damage and disease. The polyol pathway is one of metabolic pathways that converts glucose into sorbitol under the actions of the AR and NADPH. Under hyperglycemia, AR becomes activated, resulting in an elevated production of sorbitol. Subsequently, sorbitol is oxidized to fructose in the presence of sorbitol dehydrogenase (SDH) and NAD. The accumulation of fructose, the end product, can also trigger oxidative stress and subsequent tissue damage. As SDH activity remains consistent, the produced sorbitols accumulate within cells [119]. This accumulation causes an increase in cell membrane permeability, leading to the leakage of intracellular components, such as inositol and reduced GSH, ultimately triggering an oxidative stress response within the cell [120]. 

The elevation in sugar levels results in a surge in the amount of glucose metabolized through the polyol pathway. The activation of AR is contingent upon NADPH. However, the metabolism of excess glucose depletes NADPH, causing a decline in GSH production and a reduction in the scavenging capacity of ROS [121]. Consequently, this imbalance leads to a redox imbalance in vivo. Notably, the AR content in the renal cortex of diabetic patients was significantly higher compared to non-diabetic patients [122]. The polyol pathway damages the kidney as it results in an increased utilization of NADPH, which is a critical cofactor required for the oxidation of reduced glutathione [123]. Glutathione, in its reduced form, is a significant antioxidant that combats ROS. A previous study revealed that inhibiting AR improved renal function and reduced oxidative stress, inflammation, and fibrosis in rats with DKD by activating the Nrf2 pathway, suppressing the activation of the NF-κB pathway, and inhibiting the NLRP3 inflammasome [124]. It appears that by inhibiting this pathway, the progression of DKD can potentially be delayed.

#### 3.3.4. Activation of the Protein Kinase C (PKC) Pathway

Normally, the PKC in renal tissue remains inactive. However, upon hyperglycemia, the intracellular diacylglycerol content significantly increases, leading to the activation of the PKC pathway [125] (Figure 2). Moreover, PKC can also be activated indirectly through AGE/RAGE and polyol pathways [126]. PKC activation and heightened oxidative stress can regulate the expression of various DKD-related pathogenic genes [127]. This regulation can lead to a downregulation of NOS, a key factor in maintaining vascular endothelial relaxation, and an upregulation of endothelin 1, which contributes to vascular endothelial contraction. Moreover, PKC activation can also enhance the expression of collagen fibers and proinflammatory factors, ultimately expediting renal fibrosis [128,129]. This process is facilitated by transcription factor β, plasminogen activator inhibitor 1 (PAI-1), and NF-κB. The protein Klotho, with its anti-aging properties, plays a pivotal role in safeguarding the kidney. It is primarily present in the kidney and secreted into the bloodstream. Klotho deficiency was found to exacerbate diabetes-induced proteinuria and podocyte injury through the activation of PKCα, which triggered the generation of ROS [130]. Once PKCα is activated, it can further increase NADPH oxidase activity, leading to an increased production of ROS by endothelial and mesangial cells [131]. The accumulation of ROS then results in additional podocyte damage, ultimately leading to increased proteinuria levels. Therefore, it is evident that the activation of PKC plays a vital role in the pathogenesis of DKD through its effect on NADPH oxidase activity and oxidative stress. 

### 3.4. The Downstream Signaling of Oxidative Stress

#### 3.4.1. NF-κB Pathway

In normal circumstances, NF-κB is ubiquitously present in various tissue cells, maintaining an inactive state. The activation of the NF-κB transcription factor is a hub for various processes, including immunity, inflammation, cell development, growth, and survival. A substantial amount of glucose can efficiently activate toll-like receptor 4 (TLR4) and NF-κB, ultimately resulting in the production of proinflammatory mediators. The activation of the NF-κB transcription factor is triggered by various stimuli, including cytokines, ionizing radiation, and oxidative stress [132] (Figure 2). NF-κB pathway activation results in the translocation of effector molecules into the nucleus and elevated expression of proinflammatory genes, including TNF-α, MCP-1, IL-6, and TGF-β1 [13], contributing to apoptosis and necrosis in cells and tissue fibrosis, which accelerates the development of DKD [133]. Multiple studies have demonstrated that the development of DKD is intricately linked to the hyperactivation of NF-κB. It was observed that chronic treatment with pyrrolidine dithiocarbamate (PDTC), a NF-κB inhibitor, effectively blocked the activation of renal TLR4, NF-κB, and IL-6, without interfering with blood glucose levels in rats with STZ-induced DKD [134]. Through the administration of PDTC to these rats, the inhibition of NF-κB prevented renal oxidative stress by modifying the expression of HO-1 and SOD2 [134]. Notably, the suppression of NF-κB significantly mitigated renal inflammation and interstitial fibrosis [135]. NF-κB is not only a target of ROS, but also plays a significant role in bridging oxidative stress and inflammation. Nevertheless, targeting NF-κB for the treatment of DKD can be challenging due to its pervasive presence in numerous cell types.

#### 3.4.2. TGF-β Pathway

TGF-β is a multifaceted cytokine that has been established as a pivotal regulator in the development of DKD. It is widely recognized that TGF-β is secreted in a latent form, but it acquires its active state upon release from the latency-associated peptide (LAP). In both patients and animal models of DKD, the TGF-β ligands, type 2 transmembrane receptor (TGFBRs), and downstream signaling molecules, such as Smad2 and Smad3, were profoundly upregulated or activated within glomeruli, tubules, and the tubulointerstitium [136]. DKD is linked to various pernicious environmental factors, including elevated blood glucose, AGEs, hypertension, and lipid imbalances, which can trigger TGF-β signaling, either directly or indirectly through TGF-β-dependent mechanisms. Indeed, hyperglycemia, angiotensin II (Ang II), AGEs, and hyperlipidemia (specifically palmitate) have the potential to trigger the overproduction of ROS [137]. ROS has the ability to activate activated protein-1 (AP-1), a transcription factor that promotes the expression of TGF-β1. Modifying the AP-1 binding site within the TGF-β1 promoter or pharmacologically decreasing the ROS levels can effectively reduce TGF-β1 production in mesangial cells [138]. In addition, the overactivation of AP-1 may also result in an increase in the tissue inhibitor of metalloproteinase-1 (TIMP-1), which subsequently exacerbates the progression of renal fibrosis [139]. Inducing TIMP-1 expression by activating the AP-1 signal pathway also contributes to the pathogenesis of hepatic fibrosis [140]. Furthermore, oxidative stress triggers the TGF-β1 signaling pathway (Figure 2), leading to the secretion of intracellular signals, such as protein kinases or cytokines [136]. Therefore, targeting TGF-β signaling represents a promising therapeutic approach for the treatment of DKD. 

TGF-β signaling plays a vital role in renal inflammation. TGF-β can trigger the activation of NLRP3 inflammasomes, which is a key process in the production and secretion of inflammatory cytokines, such as interleukin-1β (IL-1β) and interleukin-18 (IL-18), in a Smad3-dependent manner [141]. Furthermore, NLRP3 enhances TGF-β signaling by promoting the TGF-β1-induced phosphorylation of Smad3 during the process of EMT [142]. These signals promote the accumulation of ECM and initiate the process of EMT. Consequently, this results in renal interstitial fibrosis and glomerulosclerosis. The inhibition of NLRP3 and IL-1β was found to mitigate kidney damage in mouse models with elevated mitochondrial ROS production [143], which might represent a potential therapeutic strategy for the treatment of DKD. MCC950 was found to effectively hinder the activation of the NLRP3 inflammasome and the subsequent release of interleukin-1β, leading to a significant reduction in mortality rates in mice with cryopyrin-associated periodic syndrome, a condition caused by abnormal NLRP3 inflammasome activation [144]. Similarly, the compound CY-09 successfully inhibited the assembly and activation of the NLRP3 inflammasome by blocking its ATPase activity [145]. This intervention prevented neonatal lethality in a mouse model of cryopyrin-associated periodic syndrome and also improved the metabolic characteristics in a mouse model of type 2 diabetes. Pharmacological IL-1R antagonism effectively prevented and even reversed the development of diabetic nephropathy in mice [143]. This finding highlights the potential of targeting the IL-1R signaling pathway as a therapeutic approach for the treatment of DKD.

TGF-β1 can be used as a biomarker for the early detection of fibrosis in DKD. The profibrotic effects of TGF-β1 are modulated by diverse signaling mechanisms, particularly the MAPK pathway. The MAPK family consists of three essential members, p38-MAPK, JNK, and ERK, all of which are closely linked to the progression of DKD. Once activated, p38-MAPK targets TGF-β1 and promotes the phosphorylation of Smad2/3, leading to increased expressions of fibronectin, collagen I, and collagen IV (Col-I/IV) [146]. The hyperactivation of p38-MAPK further intensifies oxidative stress in the body, ultimately leading to an increase in oxygen radicals [147]. The enzyme renalase can effectively mitigate renal interstitial fibrosis by suppressing TGF-β1-induced tubular EMT through the inhibition of the ERK pathway [148]. A separate study highlighted that, once ERK signaling was activated, it triggered the transcriptional regulation of hypoxia inducible factor-1a (HIF-1a), which subsequently modulated the levels of fibronectin, Col-I, CTGF, and epidermal growth factor receptor (EGFR) [149].

Latent TGF-β1, as opposed to its active counterpart, serves a protective role in DKD. Mice with a transgenic overexpression of latent TGF-β1 were effectively shielded from the progression of renal inflammation and renal fibrosis in models of obstructive nephropathy and crescentic glomerulonephritis [150]. However, when latent TGF-β1was overexpressed in the epidermis, it leaded to a significant activation of TGF-β/Smad signaling, which triggered inflammatory skin lesions locally, accompanied by the infiltration of numerous immune cells [151]. Therefore, clinical treatment utilizing antibody-based strategies must be approached with caution, as the majority of TGF-β1 circulating in the bloodstream exists in its latent form. Administering an anti-TGF-β antibody has the potential to negate the beneficial impact of latent TGF-β1, potentially leading to adverse outcomes in patients suffering from DKD.

#### 3.4.3. Phosphoinositide 3-Kinase (PI3K)/Akt Pathway

The PI3K/Akt pathway is primarily involved in regulating cell proliferation, differentiation, and apoptosis. PI3K triggers Akt’s translocation to the plasma membrane and subsequently activates Akt [152]. A meta-analysis showed that single nucleotide polymorphisms (SNPs) in the PI3K/Akt pathway, specifically at eNOS rs1799983, rs869109213, rs2070744, and IL-6 rs1800796, were associated with an elevated risk of developing DKD [153]. After an intensive investigation, several genes that were closely related to the basement membrane, including collagen alpha-1/2(IV) (*COL4A1*, *COL4A2*), collagen alpha-2/3(VI) (*COL6A2*, *COL6A3*), fibronectin (*FN1*), and laminin subunit beta 1 (*LAMB1*), were identified [154]. Inflammatory infiltration analysis suggested that these genes played a significant role in inflammatory processes through the PI3K/Akt signaling pathway and the AGE/RAGE signaling pathway in diabetic complications. Once Akt is activated, it starts to function by regulating downstream signaling molecules, such as glycogen synthase kinase 3β (GSK 3β), mammalian target of rapamycin (mTOR), and forkhead box protein O1 (FoxO1) [155,156].

Urinary and intra-renal GSK3β activity exfoliated cells were reported to predict the progression of DKD [157]. Additionally, hyperglycemia can cause damage to the ultrastructure of renal tissue by inhibiting the level of phosphorylated-AKT (P-AKT), and increasing the levels of P-GSK-3β, B-cell lymphoma/leukemia 2-associated X protein (BAX), and cleaved-caspase-3 in STZ-diabetic rats [156]. In animal experiments, it was observed that P-GSK3β promoted NF-κB function, leading to an increase in inflammation in DKD mice [158]. Pretreating with roxadustat, an inhibitor that targets the prolyl hydroxylase of HIF and serves as an antioxidant agent, effectively mitigates folic acid-induced kidney injury by inhibiting ferroptosis through the Akt/GSK-3β/Nrf2 signaling pathway [159]. The activation of GSK3β effectively mitigated kidney injury induced by diabetes in several studies [160].

Once Akt is activated, it phosphorylates and thereby activates mTOR, which in turn stimulates various downstream signaling cascades that are involved in cell proliferation, apoptosis, glucose metabolism, and survival. Studies revealed that ROS induced the EMT of renal tubular epithelial cells, which promoted renal fibrosis via the TGF-β1/PI3K/Akt/mTOR pathway in DKD [161] (Figure 2). Additionally, the hyperactivation of mTOR complexes (mTORC1) may contribute to the pathogenesis of podocyte damage in DKD, leading to proteinuria and tubular cell injury that can diminish renal function [162]. By selectively targeting mTORC1 and mTORC2 in renal cell populations, as well as through pharmacologic mTOR inhibition, researchers have gained a deeper understanding of the core role of mTOR in podocyte homeostasis and tubular transport. These studies have significantly contributed to our comprehension of the mechanisms involved in the development and progression of DKD. 

The PI3K/Akt signaling cascade phosphorylates FoxO1, effectively inactivating it through promoting its nuclear translocation, which is particularly vital in the development of diabetic kidney injury [163]. Accumulating evidence suggests that FoxO1 is integral to the regulation of various cellular processes, including resistance to oxidative stress, inflammation autophagy, cell cycle arrest, and apoptosis upon high glucose stimulation [164]. These findings indicate that FoxO1 plays a significant role in the pathogenesis of DKD. Furthermore, the FoxO1-mediated inhibition of STAT1 can effectively mitigate tubulointerstitial fibrosis and tubule apoptosis in DKD [165]. Moreover, the overexpression of FoxO1 is highly effective in preventing podocyte apoptosis and renal damage caused by high glucose levels, which is dependent on the expression of phosphatase and tensin homolog-induced putative kinase 1 (PINK1) [166]. Additionally, the methylation of FoxO1 is also linked to the regulation of inflammation. Hypomethylation in the promoter region of the FoxO1 gene may be associated with the development of type 2 DKD [167]. Placenta-derived mesenchymal stem cells (p-MSCs) have been found to significantly enhance renal function and reduce podocyte injury in rats with DKD by regulating the sirtuin 1 (SIRT1)/FoxO1 pathway and promoting autophagy in the podocytes [168]. These findings highlight the potential of FoxO1 as a therapeutic target for the treatment of this debilitating condition.

#### 3.4.4. Nrf2/ARE Signaling Pathway

Nrf2 is a transcription factor that holds a core function in the body’s ability to withstand oxidative stress. Nrf2 plays a pivotal role in the regulation of anti-inflammatory, antioxidant, anti-apoptotic, and anti-fibrotic processes. Moreover, it is intricately linked to the severity of diabetic kidney injury. In its normal physiological state, Nrf2 is found in the cytoplasm in an inactive state, bound to its inhibitor, Kelch-like ECH-associated protein 1 (KEAP1). However, the oxidative stress caused by high glucose disrupts the binding of Nrf2 to its inhibitor, KEAP1, leading to its activation and subsequent nuclear translocation (Figure 2). Once activated, Nrf2 binds to the antioxidant response elements (AREs) in the nucleus, effectively reducing oxidative damage and inhibiting the expression of TGF-β1, extracellular matrix proteins, and p21 [169,170]. This suppression then slows the progression of kidney injury in DKD. 

Animal studies also revealed that activated Nrf2 signaling boosted the expression of antioxidant enzymes, such as HO-1 and NQO-1 [171]. Studies also demonstrated that enhancing Nrf2 expression and impeding TGF-smad signal activation effectively mitigated glomerulosclerosis and tubular vacuolar degeneration, as well as the accumulation of fibronectin and collagen IV in the glomeruli [172]. Additionally, the upregulation of the Nrf2/HO-1 axis diminished renal levels of TNF-α, IL-6, cleavage of caspase-3, Bax, and phosphorylation of p38, effectively suppressing JNK phosphorylation and NF-κB p65 transactivation, while simultaneously increasing Bcl-2 expression [173]. Sirtuin-1, a nicotinamide adenine dinucleotide (NAD+)-dependent protein deacetylase, plays a significant role in the pathogenesis and development of DKD. The addition of AGEs to glomerular mesangial cells indicated that SIRT1 activation could reduce the production of fibronectin and TGF-β1 by stimulating the Keap1/Nrf2/ARE pathway [174]. The SIRT1/Nrf2/ARE signaling pathway effectively mitigated oxidative stress and kidney damage by curbing hyperglycemia-induced superoxide overproduction in db/db mice [175]. These findings demonstrate the anti-inflammatory and anti-apoptotic properties of activated Nrf2, which are beneficial for preventing STZ-induced DKD. In conclusion, targeting Nrf2 is a promising therapeutic approach for increasing antioxidants in DKD. However, more research is needed to fully understand its mechanisms and to develop more effective and safer treatment strategies for DKD.

#### 3.4.5. Janus Kinase 2-Signal Transducer/Activator of Transcription 3 (JAK2/STAT3) Signaling Pathway

When the specific cell-surface receptor protein encounters its extracellular ligand (such as a cytokine), JAK proteins are activated, leading to the phosphorylation of the receptor. This phosphorylation process triggers the activation of STAT, which is then phosphorylated by the receptor. The activated JAK/STAT signaling pathway is intricately involved in the regulation of cell growth and proliferation. The disruption of this pathway occurs in many diseases. The inhibition of the JAK2/STAT3 pathway can effectively protect nephrocytes and reduce renal tissue injury in rats with DKD [176]. Additional studies also revealed that the silencing of histone deacetylase 9 (HDAC9) mitigated glomerulosclerosis, inflammatory cytokine release, podocyte apoptosis, and renal injury, all of which were achieved through the JAK2/STAT3 pathway [177]. In addition, the inhibition of JAK2/STAT3 effectively alleviated the renal structural damage caused by diabetes, reduced oxidative stress, and mitigated the nuclear translocation of NF-κB and the expression of cleaved caspase-3 [178]. This intervention not only enhanced the antioxidant defense system, but also downregulated the activity of apoptotic enzymes (caspase-3/caspase-8/caspase-9) in the diabetic kidney. Studies revealed that IL-6 could exhibit a notable function in the local activation of JAK2/STAT3, leading to podocyte hypertrophy under hyperglycemic conditions [179]. Furthermore, the JAK2/STAT3 pathway can also activate macrophages in the kidney, ultimately exacerbating kidney injury by secreting inflammatory factors and generating ROS [180]. AGEs can trigger apoptosis and inflammation in mouse podocytes by activating the JAK2/STAT3 pathway via C-X-C motif chemokine ligand 9 (CXCL9) [181]. The JAK2/STAT3 signaling pathway plays a pivotal role in the regulation of immune response, inflammation, oxidative stress, and cell apoptosis during the development of DKD. 

#### 3.4.6. Adenosine Monophosphate-Activated Protein Kinase (AMPK) Signaling Pathway

AMPK serves as an energy sensor, whose aberrant expression is associated with various diseases, including cancer, cardiovascular diseases, and diabetic kidney injury. The activity of AMPK was found to be reduced in the kidneys of both diabetic mice and humans. In a high glucose condition, AMPK phosphorylation is hindered, resulting in an upsurge in peroxisome proliferator-activated receptor α (PPAR-α) expression, lipid accumulation, apoptosis, and the expression of proinflammatory and profibrotic genes [182]. Notably, the levels of AMPK, phosphorylated AMPK (p-AMPK), and SIRT1 were significantly reduced in both the DN mice and the podocyte model. Inhibiting the protein levels of TNF-α, IL-6, IL-1β, cleaved IL-1β, NLRP3, and cleaved caspase-1 in the APMK/SIRT1/NF-κB pathway effectively blocks oxidative stress and inflammatory responses associated with pyroptosis, thereby preventing the progression of DKD [183]. Furthermore, treatment with the AMPK activator triggered the production of mitochondrial superoxide, enhanced mitochondrial function, and was associated with a reduction in glomerular matrix and albuminuria in the diabetic kidney [184]. The upregulation of AMPK not only enhances insulin sensitivity, but also effectively inhibits stress and cell death in β cells, ultimately improving nephropathy. The desired effect is achieved by interacting with other molecular pathways, including peroxisome proliferator-activated receptor gamma coactivator 1-alpha (PGC-1α), PI3K/Akt, Nox4, and NF-κB [185]. The AMPK agonist had been shown to effectively mitigate renal oxidative stress and tubulointerstitial fibrosis in diabetic mice induced by a high-fat diet and STZ through activating mitophagy through a p-AMPK-Pink1-Parkin pathway [186]. The modulation of AMPK activity has been proposed as a potential therapeutic strategy for various kidney diseases. The beneficial effects of AMPK activation may be achieved through various mechanisms, including the attenuation of oxidative stress, and the regulation of autophagy. However, further research is necessary to overall understand the precise role of p-AMPK in nephropathy and its therapeutic potential in kidney disease management.

## 4. Oxidative Stress and Epigenetic Modifications

High concentrations of blood glucose can lead to epigenetic modifications, which are stable alterations in the cell phenotype that are linked to modifications in gene expression, without altering the DNA sequence itself. Poorer control of blood glucose levels was closely associated with DKD, and previous research showed that the disease might persist, even if strict glucose control is achieved, which was attributed to “metabolic memory” and epigenetic modifications [187]. These epigenetic modifications encompass DNA methylation, histone modification, and the expression of diverse types of non-coding RNAs (ncRNAs). Epigenetic modifications play a pivotal role in DKD-related processes, including inflammation, oxidative stress, hemodynamics, fibrosis, and the activation of abnormal signaling cascades, which ultimately lead to the deterioration of DKD [188]. The interaction between epigenetic modifications and oxidative stress has increasingly been recognized as an important factor in the development and progression of various diseases.

### 4.1. DNA Methylation

DNA methylation, the most stable epigenetic modification, involves the addition of a methyl group to a cytosine-phosphate-guanine (CpG) site on DNA. In general, the methylation of gene promoters does indeed effectively silence gene transcription. The epigenetic regulatory mechanisms are integral to several vital renal cell types, including mesangial cells, podocytes, tubular epithelia, and glomerular endothelial cells [189]. 

The abnormal DNA methylation of TGF-β1, caused by an overproduction of ROS, is prominent in the development of mesangial fibrosis during the progression of DKD [190]. This process can be effectively blocked by the use of antioxidants. The epigenetic modifications, including DNA methylation and histone methylation, potentially modulated the expression of Nrf2 [191]. Multiple reports have emerged discussing the indispensable functions of histone-modifying enzymes and DNA methylation patterns in preserving podocyte integrity, particularly in diabetic circumstances [192]. Kruppel-like factor 4 (KLF4), which is expressed in podocytes, has been observed to decrease in glomerular diseases, leading to the methylation of the nephrin promoter, decreased nephrin expression, and the development of proteinuria. ARBs can reset the podocyte epigenome by regulating KLF4, thereby reducing proteinuria [193]. In a methylome-wide association study of the Korean population, three methylated sites, including *COMMD1*, *TMOD1*, and *FHOD1*, were identified and found to be associated with DKD [194]. DNA methylation was associated with renal fibrosis, which was the ultimate pathophysiological stage in the development of ESRD [195]. These findings suggest that demethylating agents may hold potential as therapeutic agents in preventing the development and progression of DKD.

### 4.2. Histone Modifications

The histones serve as “spools” for DNA that interact with DNA and facilitate the formation of nucleosomes, the basic building blocks of chromatin. Multiple enzymatic reactions can modify histones, including methylation, acetylation, phosphorylation, and ubiquitination. Among these modifications, methylation and acetylation have been the most extensively studied. The methylation of histones can either activate or repress transcription, depending on the number and location of the methyl groups [196]. The histone methyltransferase enzyme EZH2, a homolog of the enhancer of the Zeste protein, plays a protective role against podocyte oxidative stress, proteinuria, and renal injury in diabetes [197]. Protein arginine methyltransferase 1 (PRMT1), which is the primary enzyme responsible for the asymmetric arginine methylation of histone proteins, plays a pronounced function in renal fibroblast activation and the development of renal fibrosis by activating the TGF-β/Smad3 signaling pathway [198].

Histone acetylation (HAc), a key epigenetic mechanism, is believed to be intricately linked to the development and progression of DKD in several cellular processes, including fibrosis, inflammation, hypertrophy, and oxidative stress [199,200]. The activation of histone acetyltransferase (HAT) p300/CBP has been observed to enhance ROS production by mediating the upregulation of NADPH oxidase, inflammation, extracellular matrix protein production, and fibrosis in diabetic mice [201]. SIRT1 deacetylase preserves energy balance during fasting by directly stimulating hepatic gluconeogenesis and mitochondrial fatty acid β-oxidation genes [202]. Resveratrol, a sirtuin-1 agonist, effectively mitigates excessive ROS production and cell death in podocytes exposed to a high-glucose environment [203]. Valproate, a histone deacetylase (HDAC) inhibitor, was found to mitigate proteinuria, podocyte damage, and renal injury by promoting autophagy and inactivating the NF-κB/iNOS signaling pathway in diabetic rats [204]. In DKD, histone H3 serine 10 phosphorylation induced the upregulation of glomerular endothelial vascular cell adhesion protein 1 (VCAM-1) [205]. Additional studies also revealed that the oxidized lipids, particularly 12(S)-hydroxyeicosatetraenoic acid (12(S)-HETE), resulting from 12/15-lipoxygenase (12/15-LOX) regulated histone modifications that were associated with the expression of profibrotic genes in mesangial cells (MCs) [206]. 

The regulation of oxidative stress in DKD is critically dependent on histone modifications. To gain an exhaustive understanding of this process and advance effective treatment options, it is imperative to conduct in-depth research that delves into the intricate mechanisms of histone modifications in DKD. Only by unravelling these mechanisms can we hope to offer patients improved outcomes.

### 4.3. Non-Coding RNAs

The non-coding RNAs (ncRNAs), including microRNAs (miRNAs), long ncRNAs (lncRNAs), and circular RNAs (circRNAs), play pivotal roles in various cellular processes, including inflammation, fibrosis, oxidative stress, and apoptosis, all of which are implicated during DKD. Incorporating the latest technological advancements, including whole-genome sequencing, single-cell sequencing, and bioinformatics, has enabled epigenetic research to become more sophisticated, leading to a wealth of data at the epigenome level. It is anticipated that these technological advancements will foster new perspectives in the diagnosis and treatment of DKD, thereby furthering our understanding of this complex condition. 

MiRNAs are endogenous ncRNAs that contain 22 nucleotides and regulate translation by binding to target mRNAs. This interaction results in the suppression of protein synthesis, providing a mechanism for fine-tuning gene expression. MiRNAs are involved in various processes that are critical to the development and progression of DKD, including fibrosis, inflammation, hypertrophy, autophagy, ER stress, oxidative stress, insulin resistance, and podocyte injury [207]. Furthermore, miRNA exerts regulatory effects on the renin-angiotensin system, AGE/RAGE signaling, and oxidative stress in diabetic nephropathy [208]. Serum exosomes from patients with diabetic kidney disease can trigger pyroptosis, stimulate the release of proinflammatory cytokines, elevate ROS levels, and enhance oxidative stress, all of which are through the mediation of miRNAs [209]. A study showed that the overexpression of miR-485 in human mesangial cells alleviated the high glucose (HG)-induced production of ROS and MDA, upregulated SOD, and attenuated inflammation and proliferation of mesangial cells in an in vitro model of diabetic nephropathy by targeting Nox5 [210]. These findings suggest that miRNAs may serve as potential therapeutic targets for the treatment of DKD. However, further research is required to confirm its efficacy and safety in vivo, as well as to fully comprehend its mechanisms of action. 

lncRNAs can bind to specific miRNAs, which subsequently enhance the expression of genes that would normally be repressed by the miRNAs. This interaction serves to mitigate the influence of these miRNAs, allowing for a more balanced gene expression profile. The abnormal expression and function of lncRNAs in key kidney cells, including mesangial cells, endothelial cells, podocytes, and tubular epithelial cells, play a pivotal role in the development of DKD [211]. For instance, brain-enriched lncRNA-containing gene 1 (Blnc1) promotes inflammation, oxidative stress, and renal fibrosis in diabetes nephropathy by regulating the Nrf2/HO-1 and NF-κB pathways [212]. LncRNAs and miRNAs have the ability to target multiple genes, making them highly versatile regulators of gene expression. LncRNA nuclear enriched abundant transcript 1 (NEAT1) enhances the proliferation, fibrosis, and EMT of mesangial cells, while simultaneously impeding their apoptosis through sponging miR-23c [213]. Additionally, in SV40 mesangial cells, lncRNA *NEAT1* can serve as a molecular “sponge” for miR-423-5p, regulating glioma pathogenesis-related 2 (*GLIPR2*), a target gene of miR-423-5p. LncRNA *NEAT1* promotes proliferation, oxidative stress, inflammation, and fibrosis while suppressing apoptosis through the miR-423-5p/*GLIPR2* axis [214]. 

Certainly, lncRNA can also provide protection during disease progression. However, the specific mechanism of lncRNA remains to be further explored. Interestingly, antisense-to-RNA-2 (CTBP1-AS2) can ease the oxidative stress, ECM accumulation, and inflammation caused by high levels of HG in diabetic nephropathy by regulating the miR-155-5p/FoxO1 axis [215]. In addition, the upregulation of lncRNA cancer susceptibility candidate 2 (CASC2) has been found to effectively suppress HG-induced proliferation, ECM accumulation, and oxidative stress in human mesangial cells (HMCs) through the miR-133b/FOXP1 regulatory axis [216]. Therefore, an in-depth exploration of the functions and mechanisms of lncRNA can help us gain a deeper understanding of the mechanisms of disease occurrence and development, providing new ideas and approaches for disease prevention and treatment.

Circular homer scaffold protein 1 (circHOMER1) enhances HG-induced oxidative stress, inflammation, and ECM accumulation in HMCs by regulating the miR-137/SOX6 axis [217]. Similarly, circ_0000491 promotes apoptosis, inflammation, oxidative stress, and fibrosis in HG-induced mesangial cells by regulating the miR-455-3p/high-mobility group box 1 (Hmgb1) axis [218]. CircRNA collagen alpha-2(I) (circCOL1A2) exerts a promotive effect on HG-induced oxidative stress and pyroptosis in diabetic nephropathy by regulating the miR-424-5p/serum and glucocorticoid-regulated kinase 1 (SGK1) axis [219]. Notably, silencing circ_0000064 alleviated HG-induced oxidative stress, inflammation, and ECM accumulation in SV40-MES13 cells by upregulating miR-30c-5p/large multifunctional protease 7 (Lmp7) axis [220]. CircRNAs have the potential to serve as valuable diagnostic indicators or therapeutic targets for the treatment of DKD.

## 5. Antioxidative Therapies

**Figure 3:**
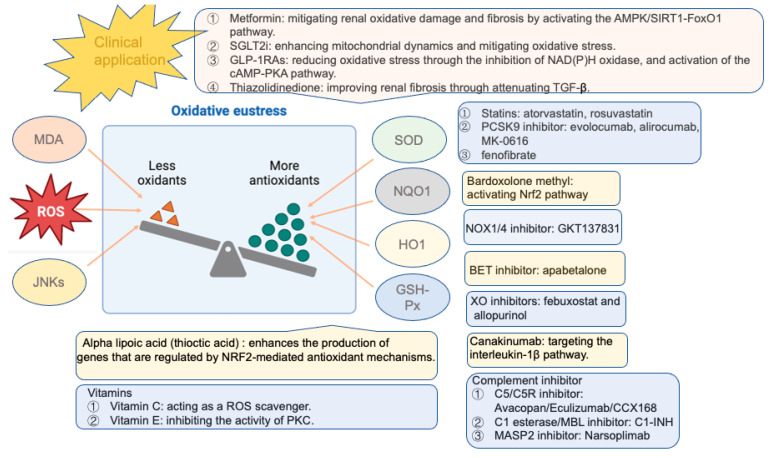
Antioxidative therapies for diabetic kidney disease. Oxidative stress can be improved by downregulating the levels of MDA, ROS, and JNKs, and elevating the expression levels of Nrf2, SOD, NQO1, HO-1, and GSH-Px. MDA: malondialdehyde; JNKs: c-Jun N-terminal kinases; SOD: superoxide dismutase; NQO1: NADPH quinone oxidoreductase; HO-1:heme oxygenase-1; GSH-Px: glutathione peroxidase; AMPK/SIRT1-FoxO1: adenosine monophosphate-activated protein kinase/sirtuin1-forkhead box protein O1; cAMP-PKA: cAMP-protein kinase A; SGLT2i: sodium-glucose co-transporter 2 inhibitor; GLP-1RAs: glucagon-like peptide-1 receptor agonists; BET: bromodomain and extra terminal; C5/C5R: complement component 5/complement component 5 receptor; C1-INH: complement component 1 esterase inhibitor; MASP-2: mannose-binding lectin-associated serine protease-2.

Oxidative stress is indeed implicated in the pathogenesis of DKD. Oxidative stress occurs when there is an imbalance between the production of ROS and the body’s ability to detoxify or neutralize them. Oxidative stress can result from hyperglycemia. High blood glucose levels can lead to overproduction of ROS and a simultaneous weakening of the antioxidant defense, further exacerbating oxidative stress. The damage caused by oxidative stress can lead to renal fibrosis, a process characterized by the accumulation of excess collagen in the kidneys. Renal fibrosis is a hallmark of DKD and is strongly associated with a decline in kidney function. Fully understanding the role of oxidative stress in DKD is paramount for developing effective therapeutic strategies. Antioxidant therapies that aim to neutralize ROS or enhance antioxidant defenses may offer a promising approach for managing DKD (Figure 3, Table 1). In this review, in addition to Nrf2 activator and glucose-lowering drugs, other novel antioxidative therapies for DKD are also investigated. However, more research is required to confirm their therapeutic potential and establish their clinical efficacy in the treatment of DKD.

### 5.1. Glucose-Lowering Drugs

As mentioned above, the damage caused by ROS can be mitigated by upregulating antioxidative enzymes, including Nrf2 signaling. Additionally, it can be addressed by decreasing ROS production, by blocking specific enzymatic reactions or, more generally, by preventing mitochondrial dysfunction.

#### 5.1.1. Metformin

Metformin, the cornerstone of pharmacological treatment of T2DM, has been established to offer potential protection against DKD by suppressing renal inflammation, oxidative stress, and fibrosis. Metformin was observed to significantly decrease serum creatinine and urine protein levels while mitigating renal oxidative damage and fibrosis in HFD/STZ-induced diabetic mice [186]. The AMPK agonist metformin effectively mitigated renal oxidative stress and tubulointerstitial fibrosis in HFD/STZ-induced diabetic mice by activating mitophagy through a p-AMPK-PTEN-induced Pink1-Parkin pathway. Additionally, metformin effectively mitigates oxidative stress and boosts autophagy in DKD by activating the AMPK/SIRT1-FoxO1 pathway [221] (Figure 3). Metformin also offers profound protection against tetrachloride and thioacetamide (TAA)-induced kidney damage and fibrosis, leading to the enhancement of the tissue-protective enzyme AMPK and the attenuation of oxidative stress, inflammation, the profibrogenic gene metalloproteinase-1 (TIMP-1), dyslipidemia, and hypertension over a period of 10 weeks in rats [222]. Metformin can also safeguard the kidneys by curbing the production of ROS and boosting mitochondrial DNA synthesis [223]. Metformin, acting as a PGC-1α activator, effectively combats kidney fibrosis by preserving mitochondrial integrity and inhibiting the NLRP3 inflammasome [224]. Additionally, metformin exerts regulatory effects on inflammation and fibrosis in DKD by targeting the tenascin-C (TNC)/TLR4/NF-κB/miRNAs inflammatory loop [225]. Nonetheless, metformin usage must be cautiously administered to patients suffering from CKD, due to the potential risk of developing lactic acidosis.

#### 5.1.2. Thiazolidinedione

Thiazolidinediones, peroxisome proliferator-activated receptor-gamma (PPAR-γ) activator, are compounds that enhance insulin sensitivity, effectively reducing plasma glucose levels and improving the lipid profile of individuals with type 2 diabetes [232]. In an in vitro study, troglitazone was demonstrated to effectively mitigate HG-induced EMT and the dysfunction of SGLTs through the modulation of the PI3K/Akt signaling pathway, which in turn regulated GSK-3β, Snail1, and β-catenin in renal proximal tubule cells [226]. Rosiglitazone was also revealed to effectively suppress calcium oxalate crystal binding and oxalate-induced oxidative stress in renal epithelial cells by promoting the activation of PPAR-γ, which subsequently regulated the expression of TGF-β1 and HGF [227]. Additionally, rosiglitazone could induce the expression of PGC-1α, finally exhibiting protective effects against oxidative stress, glomerulosclerosis, and tubulointerstitial fibrosis in an experimental model of DKD [275]. This suggests that PGC-1α may serve as a potential therapeutic target in DKD. The oral administration of the PPAR-γ agonist pioglitazone significantly attenuated TGF-β1-induced renal fibrosis by suppressing the activation of early growth response factor-1 (EGR-1), STAT3, and AP-1, thereby exerting a protective effect on kidney health [228]. Furthermore, pioglitazone was found to inhibit transient receptor potential channel C6 (TRPC6)-induced glomerular injury and proteinuria in podocytes [229]. In the present study, the researchers demonstrated that sildenafil, a PDE5 inhibitor, effectively alleviated podocyte injury and prevented proteinuria by facilitating PPAR-γ-mediated inhibition [229]. However, a retrospective cohort study reported that pioglitazone did not reduce the risk of composite renal endpoints and albuminuria [230]. Conclusive evidence regarding the effects of pioglitazone requires further randomized controlled studies to establish its definitive impact. Furthermore, the PPAR-γ agonist rosiglitazone and PPAR-γ overexpression exhibited protective effects against podocyte injury by mitigating mitochondrial dysfunction (MtD) and oxidative stress, as demonstrated by a reduction in ROS production, preservation of mitochondrial morphology, restoration of mitochondrial DNA (mtDNA) copy number, attenuation of mitochondrial membrane potential loss, and restoration of mitochondrial electron transport function [231]. A RCT indicated that rosiglitazone effectively reduced albuminuria among patients with type 2 diabetes [232]. Another RCT also revealed that rosiglitazone improved proteinuria and NO bioavailability [233]. During the study, the rosiglitazone treatment was generally well tolerated, and the major adverse event, which was the development of edema, could be effectively managed through dose adjustments of the study drug as well as the administration of diuretic agents. Nevertheless, thiazolidinediones may increase the risk of heart failure in diabetic patients [276]. These findings highlight the therapeutic potential of PPAR-γ agonists in the management of renal diseases. Therefore, further studies are necessary to better understand the mechanisms of these PPAR-γ agonists in the management of DKD. 

#### 5.1.3. SGLT2 Inhibitors

SGLT2 inhibitors have been demonstrated to possess both anti-inflammatory and oxidative stress-reducing properties, which extend beyond their impact on blood glucose levels. SGLT2 inhibitors may offer renal protection by enhancing mitochondrial dynamics and mitigating oxidative stress [223] (Figure 3). High glucose enhanced the production of ROS and elevated the expression of RAGE in tubular cells, both of which were partly mitigated by the application of SGLT2 small interfering RNAs (siRNAs) [277].

Tofogliflozin, a highly specific inhibitor of SGLT2, exerts anti-inflammatory and antifibrotic effects on experimental diabetic nephropathy partly by curbing the formation of AGEs and oxidative stress in the kidney [17]. Additionally, dapagliflozin reverses the increased expression of circRNAs in proximal tubular epithelial cells (PTECs) treated with high glucose [278]. Dapagliflozin, whether used as monotherapy or in combination with metformin, was found to be more effective than metformin alone in mitigating renal dysfunction, enhancing renal organic anion transporter 3 expression, and promoting renal autophagy in rats with diabetes mellitus [279]. Notably, dapagliflozin monotherapy displayed superior efficacy in suppressing renal oxidative stress in these rats compared to metformin or the combination treatment. The therapeutic effect of dapagliflozin relies on its ability to hinder the secretion of IL-1β by macrophages via the ROS-NLRP3-caspase-1 pathway [280]. This observation highlights the potential of dapagliflozin as a therapeutic option for managing diabetes-related renal complications. 

A study revealed that the administration of canagliflozin at a low dose had no impact on blood glucose levels or glycosuria, yet it notably improved albuminuria and mesangial expansion in db/db mice [281]. Moreover, canagliflozin effectively counteracted the activation of PKC-NAD(P)H oxidase pathway caused by high glucose levels, as well as the resulting surge in ROS production. Notably, metformin and canagliflozin also collaboratively safeguard the kidney against the progression of DKD by inhibiting the NF-κB pathway [282]. It is worth mentioning that this protection is achieved in a non-synergistic manner. The glucose-independent findings regarding SGLT2 inhibition align with the recent findings of the CREDENCE [283] and CANVAS [284] studies, which established that the SGLT2 inhibitor canagliflozin provided renoprotection in patients with low eGFR and proteinuria, even with minimal glucose-lowering effects in individuals with type 2 diabetes.

#### 5.1.4. Glucagon-like Peptide 1 Receptor Agonists (GLP-1 RAs)

The hormone GLP-1, a member of the incretin hormone family, is responsible for stimulating insulin secretion, inhibiting glucagon release, and ultimately leading to a reduction in blood glucose levels. Produced by L cells in the distal small intestine in response to the ingestion of glucose, GLP-1 has a short half-life due to its metabolism by dipeptidyl peptidase-4 (DDP-4). GLP-1 has been demonstrated to exert renoprotective effects by interfering with the signaling and expression of RAGE [16]. Additionally, GLP-1 RAs also possess the remarkable ability to modulate immune cell signaling and regulate the NF-κB pathway [285].

DPP-4 inhibitors effectively suppressed the functional activity of the lectin pathway in a dose-dependent manner, displaying varying potency in vitro [245]. When tested in vivo, levels of MBL, sMAC, and C4b decreased significantly over time in both groups, with no significant impact observed from sitagliptin treatment. The GLP-1 receptor is expressed in glomerular capillaries, but not in tubuli [14]. Kidney outcomes have been investigated as a secondary outcome or in post hoc analyses in cardiovascular outcome trials of antidiabetic GLP1 RAs in type 2 diabetes. Diabetic mice with a GLP-1 receptor knockout exhibited elevated glomerular superoxide levels, increased renal NAD(P)H oxidase activity, and reduced cAMP and protein kinase A (PKA) activity in the kidneys [14]. These mice also exhibited more severe albuminuria and mesangial expansion compared to the wildtype diabetic controls, despite having comparable levels of hyperglycemia. These findings suggest that GLP-1 exerts conspicuous role in protecting against increased renal oxidative stress under chronic hyperglycemia. This protection is likely achieved through the inhibition of NAD(P)H oxidase, a major source of superoxide, and activation of the cAMP-PKA pathway. The trials of ELIXA (lixisenatide), EXSCEL (exenatide), LEADER (liraglutide), SUSTAIN-6 (semaglutide), and REWIND (dulaglutide) all demonstrated beneficial effects on the kidney [237,239,240,242,286]. These positive effects were likely to be at least partially attributed to improved glucose control. With its antioxidant properties, low-dose lixisenatide effectively protected against early onset nephropathy in diabetic rats [238]. This protection was evident in the significant reduction in renal MDA and total Nox levels, accompanied by a notable enhancement in total antioxidant capacity. Liraglutide was found to reduce diabetes-induced albuminuria and mesangial expansion, as well as attenuate glomerular Nox4 expression and oxidative stress [14]. Another study also revealed that liraglutide not only safeguarded the kidneys but also enhanced diabetic nephropathy (DN) by hindering inflammation and oxidative stress, mitigating urinary albumin excretion, preserving podocyte integrity, and bolstering renal function beyond its hypoglycemic attributes [241]. GLP-1 attenuated DKD through activation of autophagy by regulating the GLP-1R-AMPK-mTOR-autophagy-ROS signaling axis [287] (Figure 3). Additionally, exenatide treatment was revealed to effectively mitigate albuminuria, glomerular injury, macrophage infiltration, and reduce the protein levels of ICAM-1 and type IV collagen in kidney tissue [15]. Furthermore, it also diminished oxidative stress and NF-κB activation in a model of type 1 diabetes without altering blood glucose levels. Conversely, the Harmony Outcomes trial (albuglutide) did not observe any significant secondary renal outcomes [244]. The FLOW trial was the first study to evaluate the impact of semaglutide on kidney outcomes in individuals with both CKD and T2D [243]. The results of this trial are eagerly awaited, and it is anticipated to be completed by late 2024. 

### 5.2. NRF2 Activation

Oxidative stress can trigger conformational adjustments in Keap1, ultimately leading to the preservation of Nrf2 from proteasomal degradation and its subsequent translocation to the nucleus. In vitro studies utilizing cells derived from Nrf2 KO and Keap1 KO mice revealed that the Keap1-Nrf2 pathway exhibited a central role in regulating mitochondrial and cytosolic ROS production through the upregulation of Nox2 and Nox4, respectively [288]. Furthermore, dietary supplementation with sulforaphane or cinnamic aldehyde, compounds that targeted Nrf2, effectively mitigated diabetes-induced kidney damage, including the attenuation of urinary albumin excretion, kidney hypertrophy, and glomerular matrix accumulation, via the modulation of oxidative stress [169].

Bardoxolone methyl, an activator of Nrf2, has undergone initial testing in a phase 2 clinical trial involving patients with DKD (BEAM study), demonstrating a significant improvement in eGFR after 52 weeks of treatment [246]. However, the phase 3 RCT to assess the efficacy of bardoxolone methyl in delaying progression to ESRD and cardiovascular deaths in patients with stage 4 CKD and type 2 diabetes (BEACON) was terminated due to a higher rate of cardiovascular events [247] (Figure 3). Nevertheless, the phase 2 clinical trial of bardoxolone methyl in individuals with CKD and type 2 diabetes (TSUBAKI study) demonstrated that bardoxolone methyl significantly boosted the measured GFR with no incidence of heart failure [248], offering a potential treatment option for kidney disease patients. Unfortunately, the subsequent phase 3 study of bardoxolone methyl in patients with diabetic kidney disease (AYAME study) was terminated as it was challenging to submit a marketing authorization application for bardoxolone methyl in the treatment of DKD due to an alarming increase in early heart failure events in patients participating in the study (NCT03550443). Additionally, the CARDINAL trial of bardoxolone methyl in alport syndrome (AS) demonstrated that bardoxolone methyl was devoid of any nephron-protective effect. Instead, it was associated with significant heart, liver, and renal toxicity in patients with CKD, including those with AS [289]. Given these sobering findings, research programs could delve into the potential clinical applications of novel Nrf2 modulators, even beyond the kidney field, devoid of the toxic effects associated with bardoxolone methyl.

### 5.3. Other Novel Antioxidative Therapies

Vitamin C can also function as a ROS scavenger by donating an electron to a substrate such as O_2_^•–^ [253]. Other antioxidants, including vitamin D, vitamin E, vitamin B9, N-acetylcysteine (NAC), lipoic acid (LA), and coenzyme Q10 (CoQ10), have the potential to regulate the levels of ROS [254,255,256]. Notably, vitamin E could effectively suppress oxidative stress by inhibiting the activity of PKC [257]. Unfortunately, the trial was unable to establish any significant effect of pyridorin on the progression of serum creatinine at 1 year in type 2 diabetic nephropathy [261]. The phase 3 RCT aimed at assessing the safety and efficacy of pyridorin administered 300 mg twice daily (BID) in the treatment of nephropathy resulting from type 2 diabetes mellitus was terminated prematurely (NCT02156843). The management of these antioxidants failed to demonstrate any benefits in the treatment of diabetes and its complications in humans, mainly due to their poor solubility, permeability, stability, and specificity [290]. 

Alpha lipoic acid (ALA), also known as thioctic acid, is a vital antioxidant compound that serves as a cofactor for mitochondrial respiratory enzymes. Alpha lipoic acid (a-LA) enhances the production of genes that are regulated by Nrf2-mediated antioxidant mechanisms, as well as peroxisome proliferator-activated receptors (PPARs) [260]. A meta-analysis concluded that the combination of a-LA and valsartan significantly lowered urinary albumin levels and oxidative stress, boosted antioxidant capacity, and mitigated renal function damage in patients with diabetic nephropathy [258]. Furthermore, a RCT demonstrated that the combination of ALA with alsartan effectively reduced inflammatory cytokines in the serum and improved renal function in individuals with early stage diabetic kidney disease [259]. Currently, a phase 3 RCT aiming to investigate the potential therapeutic role of adjuvant alpha lipoic acid as an antioxidant in the treatment of diabetic nephropathy associated with type 1 diabetes is currently recruiting participants (NCT06253429). 

GKT137831, a pioneering Nox1/4 inhibitor, failed to effectively reduce albuminuria in a clinical trial (NCT02010242). Although, a significant kidney protective effect of XO inhibitors was observed in animal experiments. In a clinical trial involving type 2 diabetic patients, febuxostat did not significantly reduce albuminuria or enhance eGFR [262]. An additional trial examining the impact of febuxostat on adipokines and kidney disease in individuals with diabetic chronic kidney disease was completed, with the results pending publication (NCT01350388). The phase 3 multicenter clinical trial of allopurinol to prevent kidney function loss in type 1 diabetes (NCT02017171) and the phase 4 RCT of allopurinol on microalbuminuria in patients with type 1 diabetes (NCT02829177) were successfully completed, yet the results of both trials were forthcoming and were yet to be published. The phase 2 RCT aimed at assessing the impact of green tea extract on soluble RAGE and kidney disease in individuals with diabetes mellitus type 2 was initiated in 2018 (NCT03622762), yet the current status of the study remained unknown.

Interventions targeting IL-1β suggest that IL-1β plays a role in the pathogenesis of DKD [143]. The anti-inflammatory therapy targeting the interleukin-1β innate immunity pathway with canakinumab was found to significantly reduce the rate of recurrent cardiovascular events compared to the placebo, even without lipid-level lowering [291]. However, it also reported an increase in fatal infections, without any impact on overall mortality. Post hoc analysis of the study on IL-1β inhibition with canakinumab revealed that it neither provided clinically meaningful benefits nor posed substantive harm with respect to serial measures of eGFR, creatinine, and the uACR [263]. 

The selective bromodomain and extra terminal (BET) inhibitor apabetalone was the pioneering epigenetic regulator to complete phase 3 clinical trials in diabetic kidney disease, with the secondary endpoint being kidney function [265]. The two-part phase 2a study of RVX000222 (another BET inhibitor) in patients with end-stage renal disease undergoing hemodialysis is scheduled to commence in November, 2024 (NCT03160430). 

The activation of the complement system is a primary pathogenic mechanism in numerous inflammatory disorders, leading to extensive efforts to develop drugs that target this system. In 2007, eculizumab was the first anti-C5 inhibitor to receive FDA approval for the treatment of paroxysmal nocturnal hemoglobinuria (PNH), followed by atypical hemolytic uremic syndrome (aHUS) [292]. However, no data on eculizumab are currently available for DKD. In the context of antineutrophil cytoplasmic antibody (ANCA)-associated vasculitis (AAV), the C5aR inhibitor avacopan, whether administered alone or in combination with low-dose corticosteroids, demonstrated non-inferiority but not superiority to standard high-dose corticosteroid treatment [293]. The European League Against Rheumatism (EULAR) provides updated guidance that avacopan may be considered as part of a strategy to reduce exposure to corticosteroids in granulomatosis with polyangiitis (GPA) or microscopic polyangiitis (MPA) [294]. Multiple ongoing or planned studies assess the efficacy of C5 inhibition in treating various diseases, including hidradenitis suppurativa (NCT03852472), C3 glomerulopathy (NCT03301467), and immunoglobulin A nephropathy (NCT02384317). A separate study evaluated the impact of CCX168, a C5aR antagonist, in atypical aHUS patients with or without genetic abnormalities in the complement system or thrombomodulin. However, this study (NCT02464891) was terminated. To conduct further experiments with C5 inhibition in DKD, we require more detailed data on its long-term safety profile.

C1INH, the recombinant variant of human C1 esterase inhibitor, is marketed under the brand name Cinryze by Shire Pharmaceuticals. This protein acts as an essential regulator within the complement system, interrupting the classical pathway and managing the intrinsic coagulation cascade. Currently, it has been approved by the FDA for the treatment of hereditary angioedema [295]. A phase I/II RCT of C1INH did not lead to a reduction in dialysis sessions one week after transplantation [266]. However, at one-year post-transplant, patients treated with C1INH experienced significantly fewer dialysis sessions and exhibited significantly better renal function compared to control patients. It is noteworthy that MBL and C1q share relevant structural and functional similarities [296]. In particular, both MBL and C1q activate C4 and C2 to generate the C3 convertase C4bC2a [297]. Furthermore, both MBL and C1q are inhibited by C1INH. As such, C1INH can downregulate the lectin pathway in DKD, thus offering a new therapeutic approach. OMS721 is a monoclonal antibody that specifically targets MBL-associated serine protease-2 (MASP-2), the enzyme responsible for mediating the binding of MBL and the cleavage of C4 and C2. The treatment using narsoplimab (OMS721) was found to be safein significantly improving the markers of thrombotic microangiopathy (TMA) in the laboratory, and leading to clinical improvement and favorable overall survival in patients with hematopoietic stem-cell transplantation-associated thrombotic microangiopathy (HSCT-TMA) [298]. Currently, OMS721 (narsoplimab) is under investigation for patients with immunoglobulin A nephropathy (IgAN), lupus nephritis (LN), membranous nephropathy (MN), and component 3 (C3) glomerulopathy, including dense deposit disease (NCT02682407). Given its specific targeting of the lectin pathway, this agent offers a viable option for the treatment of DKD without causing widespread complement inhibition. 

Hyperlipidemia has been implicated as a potential factor contributing to the initiation and advancement of DKD [299]. Statin therapy is generally recommended for secondary prevention among individuals who have already been diagnosed with CVD and for primary prevention among adults over the age of 40 who have diabetes [300]. Furthermore, statin therapy is indicated for primary prevention in individuals aged 40 and above who have CKD stages 1–4 and those who have undergone kidney transplantation [300]. Additional LDL-lowering drugs include bile acid sequestrants, ezetimibe, and proprotein convertase subtilisin/kexin type 9 inhibitors (PCSK9i). The KDIGO guidelines advise patients with DKD to undergo statin therapy and non-statin therapies (ezetimibe, PCSK9i, or icosapent ethyl) as a preventive measure against cardiovascular events [6]. Additionally, a RCT revealed significant renoprotective effects of atorvastatin and rosuvastatin in patients with diabetes who had moderate proteinuria [300]. Furthermore, a phase 4 RCT to assess the potential benefits of acute pretreatment with a high dose of atorvastatin in contrast induced nephropathy patients with diabetes indicated for elective coronary intervention has been completed by 2020 (NCT04375787), and the data from this study are not publicly available. However, a cochrane database systematic review reported that there was insufficient data to definitively assess the potential benefits and harms of statin therapy in reducing the risk of death, major cardiovascular events, and myocardial infarction among individuals with CKD not requiring dialysis who did not have cardiovascular diseases at the initial evaluation [301]. 

Triglyceride-lowering drugs, especially fenofibrate, an agonist of PPAR-α, were explored in a phase 3 interventional study to analyze the state of fuel metabolism in patients with DKD (NCT03869931). Currently, the study status is unknown. PCSK9, primarily secreted by the liver, is a key regulator in maintaining cholesterol homeostasis by inducing the degradation of low-density lipoprotein (LDL) receptors. Oxidized LDL (ox-LDL) can trigger the overexpression of PCSK9, and vice versa, PCSK9 also potentiates the formation of ox-LDL by stimulating Nox2-mediated oxidation [302]. Moreover, the overexpression of the PCSK9 gene induced by plasmid transfection potentiates the production of ROS in macrophages derived from knockout mice [303]. Furthermore, SIRT3 can mediate the effects of PCSK9 inhibitors on autophagy, inflammation, and oxidative stress in endothelial cells [304]. Patients with elevated levels of PCSK9 also demonstrated a remarkably accelerated rate of ROS production and ox-LDL generation in patients with atrial fibrillation [305]. PCSK9 inhibitors effectively suppress oxidative stress and inflammation during atherosclerotic development by promoting macrophage autophagy [250]. Furthermore, the PCSK9 inhibitor alirocumab was revealed to effectively modulate oxidative stress by reducing the hepatic levels of lipid peroxidation products in a rat model of alcohol-induced liver injury [251]. Additionally, a study has demonstrated the antioxidative and cytoprotective effects of evolocumab, another PCSK9 inhibitor, against oxidative damage induced by H_2_O_2_ in human umbilical vein endothelial cells [252]. A multicenter, longitudinal study on 80 patients with heterozygous familial hypercholesterolemia (HeFH), revealed a significant reduction in oxidative stress after 6 months of treatment with PCSK9i [306]. The increasing evidence has shown an association between PCSK9 and oxidative stress, suggesting that inhibiting PCSK9 can emerge as a promising novel therapy to mitigate oxidative stress-related DKD. 

In patients with DKD, therapy with lipid-lowering drugs, particularly the combination of fibrates and statins, was independently associated with elevated PCSK9 levels [307]. PCSK9 is implicated in the induction of inflammation and activation of the cyclic GMP-AMP (cGAMP) synthase/stimulator of interferon gene (cGAS/STING) pathway in DKD [308]. Furthermore, the potential of PCSK9 levels as a biomarker in identifying DKD patients who may benefit from anti-PCSK9 strategies warrants further investigation. A study to investigate the differences in the plasma pharmacokinetic profiles of the oral PCSK9 inhibitor (MK-0616) between individuals with varying degrees of renal impairment (ranging from moderate to severe, including ESRD) receiving statin therapy and a matched control group of healthy individuals also receiving statin therapy was completed by 2024 (NCT05934292). However, the relevant date has not been made available for publication. This is essential to further elucidate the role of PCSK9 in the pathogenesis and progression of DKD, and to evaluate the potential of PCSK9-targeted therapies in the treatment of this enfeebling disorder.

Abundant evidence has demonstrated that mesenchymal stem cell (MSC) therapy is effective in protecting organs against various pathological conditions against various pathological conditions primarily by suppressing inflammation and regulating both innate and adaptive immune responses, thereby downregulating immunogenicity [267,309,310,311]. Furthermore, molecular detection revealed that MSCs possess the potential to downregulate the expression of renal fibrosis-associated markers, including TGF-β, Col-I, FN, α-SMA, and E-cadherin, along with the expression of inflammatory mediators like TNF-α and MCP-1. Stem cells are self-renewing, self-replicating pluripotent cells and can be categorized according to their origin: embryonic stem cells, adult stem cells, and induced pluripotent stem cells. Adult stem cells, which are undifferentiated cells found within differentiated tissues, can be isolated from various sources including bone marrow (BM), adipose tissue, umbilical cord blood, and deciduous teeth. 

The combination of MSCs and miR-124a effectively shielded kidney tissue from damage and suppressed nephrocyte apoptosis in DKD by inhibiting the notch signaling pathway [312]. MSC-derived small extracellular vesicles (MSC-sEVs) offer promising potential for repairing or preventing damage associated with DKD through anti-inflammatory effects, reduction in endoplasmic reticulum-related protein stress, polarization of M2 macrophages, and increasing autophagy [313,314]. Furthermore, a study also revealed that MSC-sEVs could improve DKD through the NLRP3 signaling pathway [315]. Additionally, the study indicates that MSC-EVs can alleviate mitochondrial damage and inflammation by stabilizing mitochondrial DNA [316]. The intra-renal arterial (IRA) transfusion of human umbilical cord-derived mesenchymal stem cell (HUCDMSC) therapy effectively maintained residual renal function and architectural integrity in rats with DKD [267]. Moreover, the intravenous administration of HUCDMSCs provided a significant amelioration of glomerular abnormalities and interstitial fibrosis in a mouse model of STZ-induced diabetes without affecting hyperglycemia, regardless of whether administered early or late in the disease course, which was associated with reduced circulating TGF-β1 levels and restoration of intra-renal autophagy [268]. 

P-MSCs can effectively alleviate renal damage and mitigate podocyte injury in rats with DKD by enhancing the SIRT1/FOXO1 pathway mediated by autophagy [168]. Furthermore, p-MSCs can also effectively ameliorate podocyte injury and attenuate PINK1/Parkin-mediated mitophagy inhibition in DKD through the activation of the SIRT1-PGC-1α-TFAM pathway [269]. Multiple intravenous infusions of adipose tissue-derived MSCs (ADMSCs) exert remarkable protective effects against chronic T2DM complications, effectively reducing inflammation and enhancing tissue repair mechanisms [270]. Although DKD can modify the transcriptome and function of ADMSCs, it remarkably preserves their immunomodulatory and paracrine activities crucial for renal repair [271]. Bone marrow-derived MSCs (BM-MSCs) effectively retarded DKD in male rats by regulating endoplasmic reticulum (ER) stress, oxidative stress, inflammation, and apoptotic pathways [272]. Animals treated with MSCs exhibited reduced urinary albumin-to-creatinine ratios (ACR), minimal mesangial expansion, increased podocyte counts, upregulated expression of mitochondria-related survival genes, suppressed autophagy hyperactivation, and potentially decreased caspase 3 expression, indicating improved renal function and cellular viability [317]. A RCT to assess the safety, tolerability, dosing effect, and early signals of efficacy of intra-arterially delivered autologous (from self) ADMSCs in patients with progressive DKD was terminated without explanation (NCT03840343). However, an another RCT indicated the safety and tolerability of intravenous MSC (ORBCEL-M) therapy in the patients with DKD [318]. In addition, a RCT also demonstrated the safety, tolerability, and efficacy of administering two doses of mesenchymal precursor cells in individuals with diabetic nephropathy and type 2 diabetes [273]. Currently, a RCT is recruiting participants to evaluate the therapeutic potential of MSCs for DKD (NCT04125329), with an expected completion date of 2024. These findings suggest that MSCs can emerge as a promising alternative cell-based therapy for managing long-term diabetic complications. Nevertheless, more preclinical animal studies need to be performed to verify the safety and efficacy of MSCs.

Endothelial dysfunction, a critical initial stage of vascular disease, is characterized by a decrease in the bioavailability of the essential endothelial vasodilator, NO, along with an increase in inflammation and oxidative stress [47]. Upregulating soluble guanylate cyclase and cGMP can ameliorate diabetic nephropathy through modulating NO activity [47,274]. A study revealed that soluble guanylate cyclase activator cinaciguat treatment restored the glomerular cGMP content and soluble guanylate cyclase expression, improved diabetes-induced glomerular damage, apoptosis, podocyte injury, proteinuria, and TIMP-1 overexpression by suppressing TGF-β and ERK1/2 signaling [48]. Furthermore, additional studies also demonstrated the renoprotective effects of a guanylate cyclase activator or stimulator through increasing cGMP via the NO-soluble guanylate cyclase pathway [47,319,320,321,322,323,324]. A RCT revealed that PF-00489791, an inhibitor of cGMP-hydrolyzing enzyme phosphodiesterase type 5 (PDE5), had been found to effectively reduce albuminuria in patients with overt diabetic nephropathy [274]. Moreover, hepatocyte growth factor (HGF) effectively mitigated HG-induced oxidative stress in rat mesangial cells, primarily by augmenting the nitric oxide production and subsequent elevation of 8-nitro-cGMP levels [325]. These findings underscore the potential therapeutic value of these agents targeting guanylate cyclase or cGMP in managing renal diseases.

## 6. Conclusions and Perspectives

DKD, a multifaceted disorder, is intricately linked to various factors, including oxidative stress, metabolic dysfunction, inflammation, and epigenetic changes. The brevity of effector molecules’ half-lives, particularly ROS, often results in the immediate effects of oxidative stress being confined to a narrow timeframe and limited spatial scope. Oxidative stress, a key driver in the pathogenesis of DKD, is initiated by various factors. Hyperglycemia, insulin resistance, and inflammation are some of the factors that can lead to an increase in the production of ROS, ultimately initiating oxidative stress. This excess production of ROS can inflict damage on glomerular and tubular cells, furthering the development and progression of DKD. The oxidants can interact with various molecules, leading to a wide range of pathophysiological effects. Therefore, oxidants can be considered as a central hub of the diverse pathogenic pathways that contribute to the development of DKD. Interventions aimed at reducing the production of oxidants and enhancing the production of antioxidants hold great promise as therapeutic strategies for chronic inflammatory diseases such as DKD [326] (Figure 3).

In addition to reducing blood sugar levels, metformin, SGLT2 inhibitors, and GLP-1 RAs also possess antioxidant properties that further contribute to their therapeutic benefits. Importantly, the future prospects of bardoxolone for the treatment of DKD remain uncertain. 

When patients with DKD experience macroalbuminuria, it becomes challenging to effectively halt the progression of the disease, despite the rigorous control of blood sugar and blood pressure levels. This difficulty may be partly attributed to the persistence of epigenetic modifications. Moreover, these persistent modifications also offer potential targets for innovative therapeutic strategies. The field of epigenetics has opened up new avenues for exploring the causes and pathogenesis of DKD, as well as for diagnosing and treating it. Nevertheless, our current comprehension of the role of epigenetic modifications in DKD remains primitive, particularly in relation to histone modifications. The development of miRNA-based treatments involves several critical considerations. Among these, the creation of stable, synthetic, and biodegradable carriers stands out as a vital aspect [327]. Additionally, the implementation of appropriate delivery mechanisms is crucial for ensuring the effective transportation of miRNAs to their target sites. By refining these techniques, we can better understand how epigenetic modifications contribute to DKD and potentially identify new therapeutic strategies. 

To address this, a novel approach in research and therapeutic developments for DKD could focus on enhancing the antioxidant defense system’s ability, including augmenting the activity of antioxidant enzymes, and blocking oxidative stress-related pathways.

In conclusion, oxidative stress plays a pivotal role in the development of DKD. Notably, both an excess and a scarcity of oxidants can have adverse effects on cells and organs. Therefore, it is essential to consider the timing and location when aiming to target oxidative stress, ensuring that therapeutic benefits are achieved without causing adverse effects. Given the intricate pathogenesis of DKD, it is imperative to develop innovative, multifaceted treatments that directly target oxidative stress and inflammation, eventually halting the progression of renal fibrosis. Renal fibrosis, an irreversible consequence of DKD, culminates in renal failure by way of causing renal damage, hypoxia, and apoptosis [328]. Future research efforts should concentrate on enhancing early detection methods and developing more effective combined treatment strategies for this complex and multifaceted disease.

## Figures and Tables

**Table 1:** Antioxidant therapy for diabetic kidney disease (DKD).

Target Drugs	Type of Study	Promising Effects	Reference
Glucose-lowering drugs			
Metformin	Animal studies	Ameliorating renal oxidative damage and fibrosis	[186,221,222,223,224,225]
Troglitazone	In vitro	Reducing EMT in renal tubule cells	[226]
Rosiglitazone	In vitro	Suppressing oxidative stress in renal epithelial cells	[227]
	Animal studies	Protecting against oxidative stress and renal fibrosis	[227,228]
	Animal studies	Mitigating glomerular injury and proteinuria in podocytes	[229]
	Clinical study	Exhibiting no influence on albuminuria	[230]
Rosiglitazone	In vitro/vivo	Protecting against podocyte injury	[231]
	Clinical trials	Reducing albuminuria	[232,233]
SGLT2 inhibitors			
Tofogliflozin	In vitro	Attenuating oxidative stress	[17]
Dapagliflozin	Clinical trial	Improving renal events	[234,235]
Canagliflozin	Clinical trial	Reduction in death due to renal events	[234]
Empagliflozin	Clinical trial	Lower incidence of DKD	[236]
GLP-1 RAs			
GLP-1 receptor	Animal study	Protecting against oxidative stress	[14]
Lixisenatide	Clinical trial	Reducing albuminuria (post hoc analysis)	[237]
	Animal study	Enhancing antioxidant effect	[238]
Exenatide	Clinical trial	Improving decline in eGFR (post hoc analysis)	[239]
	Animal study	Protecting against the progression of DKD	[15]
Liraglutide	Clinical trial	Lower rates of progression of DKD (secondary analysis)	[240]
	Clinical study	Reducing inflammation and oxidative stress	[241]
	Animal study	Reducing albuminuria and attenuating oxidative stress	[14]
Semaglutide	Clinical trial	Reducing the rate of eGFR decline (post hoc analysis)	[242]
	Clinical trial	Ongoing (primary renal endpoints)	[243]
Albuglutide	Clinical trial	Presenting no significant secondary renal events	[244]
DDP-4 inhibitors	Clinical trial	Modulating complement activation	[245]
Nrf2 activator			
Sulforaphane/cinnamic	Animal study	Reducing renal damage	[169]
Bardoxolone	Clinical trial	Improving eGFR	[246]
	Clinical trial	Being terminated due to cardiovascular events	[247]
	Clinical trial	Improving eGFR without heart failure	[248]
	Clinical trial	Being terminated due to high incidence of heart failure	NCT03550443
Lipid-lowering drugs			
Atorvastatin	Clinical trial	Improving proteinuria	[249]
Rosuvastatin	Clinical trial	Exhibiting renoprotective effects	[249]
Evolocumab (PCSK9i)	Animal study	Suppressing oxidative stress and inflammation	[250]
Alirocumab (PCSK9i)	Animal study	Reducing oxidative stress	[251]
Evolocumab (PCSK9i)	In vitro	Protecting against oxidative damage	[252]
MK-0616 (PCSK9i)	Clinical trial	Having been completed	NCT05934292
Fenofibrate	Clinical trial	Unknown status	NCT03869931
Vitamins	Animal study	Regulating the levels of ROS	[253,254,255,256,257]
Thioctic acid	Clinical study	Reducing urinary albuminuria	[258]
	Clinical trial	Improving renal functions in early DKD	[259]
	Clinical trial	Recruiting patients	NCT06253429
	Animal study	Exerting antioxidative properties	[260]
Pyridorin	Clinical trial	Showing no influence on serum creatinine	[261]
	Clinical trial	Being terminated with no results	NCT02156843
Nox inhibitor			
GKT137831(Nox1/4)	Clinical trial	Exhibiting no influence on albuminuria	NCT02010242
	Animal studies	Mitigating glomerular injury and proteinuria in podocytes	[229]
XO inhibitor			
Febuxostat	Animal study	Slowing the progression of albuminuria	[57]
Febuxostat	Clinical trial	Having no influence in albuminuria or eGFR	[262]
	Clinical trial	Having been completed without results	NCT01350388
Allopurinol	Animal study	Reducing albuminuria and attenuating renal injury	[56]
	Clinical trial	Having been completed without results	NCT02017171; NCT02829177
AGE/RAGE inhibitor			
Aminoguanidine (AGE)	Animal study	Reducing albuminuria and renal damage	[113]
Pyridoxamine (AGE)	Animal study	Slowing the progression of DKD	[114]
Alagebrium (AGE)	Clinical trial	Having been terminated	NCT00557518
	Animal study	Ameliorating renal damage and inflammation	[115]
RAGE antibody	Animal study	Attenuating albuminuria and renal injury	[116]
Green tea (RAGE)	Clinical trial	Without status of the study	NCT03622762
Canakinumab (IL-1β)	Clinical trial	With no adverse renal events (post hoc analysis)	[263]
Apabetalone (BET)	Clinical trial	Improving kidney function (post hoc analysis)	[264,265]
	Clinical trial	Not yet recruiting	NCT03160430
C3a/C5aR antagonist	In vitro/vivo	Ameliorating EMT	[104]
	In vitro/vivo	Modulating inflammation and metabolic function	[106]
C3aR antagonism	Animal study	Improving proteinuria and kidney function	[97]
C1INH	Clinical trial	Improving renal function	[266]
Mesenchymal stem cells			
HUCDMSCs	Animal study	Ameliorating renal fibrosis	[267,268]
p-MSCs	Animal study	Attenuating renal damage	[168,269]
ADMSCs	Animal study	Enhancing renal repair	[270,271]
BM-MSCs	Animal study	Alleviating renal damage	[272]
MSCs	Clinical trial	Being safe and tolerated for clinical use	[273]
Guanylate cyclase activator			
Cinaciguat	Animal study	Improving proteinuria	[48]
PF-00489791(PDE5i)	Clinical trial	Improving albuminuria	[274]

Antioxidant therapy targeting oxidative stress, including clinical and preclinical effects. DKD: diabetic kidney disease; EMT: epithelial-to-mesenchymal transition; SGLT2: sodium-glucose co-transporter 2; GLP-1 RAs: glucagon-like peptide-1 receptors; eGFR: estimated glomerular filtrate rate; DDP-4: dipeptidyl peptidase 4; NRF2: nuclear factor erythroid 2-related factor 2; PCSK9i: proprotein convertase subtilisin kexin 9 inhibitor; Nox: nicotinamide adenine dinucleotide phosphate oxidase; XO: xanthine oxidase; AGEs: advanced glycation end products; RAGEs: receptors for AGEs; IL-1β: interleukin-1β; BET: bromodomain and extra terminal; C3a/C5aR: C3a/C5a receptor; C1INH: C1 esterase inhibitor; HUCDMSCs: human umbilical cord-derived mesenchymal stem cells; p-MSCs: placenta-derived MSCs; ADMSCs: adipose tissue-derived MSCs; BM-MSCs: bone marrow-derived MSCs.
